# A call to action: the SHEA research agenda to combat healthcare-associated infections

**DOI:** 10.1017/ice.2024.125

**Published:** 2024-09

**Authors:** Jennie H. Kwon, Sonali D. Advani, Westyn Branch-Elliman, Barbara I. Braun, Vincent Chi-Chung Cheng, Kathleen Chiotos, Peggy Douglas, Shruti K. Gohil, Sara C. Keller, Eili Y. Klein, Sarah L. Krein, Eric T. Lofgren, Katreena Merrill, Rebekah W. Moehring, Elizabeth Monsees, Luci Perri, Felicia Scaggs Huang, Mark A. Shelly, Felicia Skelton, Emily S. Spivak, Pranavi V. Sreeramoju, Katie J. Suda, Joseph Y. Ting, Gregory David Weston, Mohamed H. Yassin, Matthew J. Ziegler, Lona Mody

**Affiliations:** 1 Washington University School of Medicine in St. Louis, St. Louis, MI, USA; 2 Duke University School of Medicine, Durham, NC, USA; 3 VA Boston Healthcare System, VA National Artificial Intelligence Institute (NAII), Harvard Medical School, Boston, MA, USA; 4 The Joint Commission, Oakbrook Terrace, IL, USA; 5 Queen Mary Hospital, Hong Kong Special Administrative Region, China; 6 Perelman School of Medicine at the University of Pennsylvania, Philadelphia, PA, USA; 7 Washington State Department of Health, Seattle, WA, USA; 8 University of California Irvine School of Medicine, UCI Irvine Health, Irvine, CA, USA; 9 Johns Hopkins University School of Medicine, Baltimore, MD, USA; 10 Johns Hopkins School of Medicine, Baltimore, MD, USA; 11 VA Ann Arbor Healthcare System, University of Michigan, Ann Arbor, MI, USA; 12 Paul G. Allen School for Global Health, Washington State University, Pullman, WA, USA; 13 Brigham Young University College of Nursing, Provo, UT, USA; 14 Duke University Medical Center, Durham, NC, USA; 15 Children’s Mercy Kansas City, University of Missouri-Kansas City School of Medicine, Kansas City, MI, USA; 16 Infection Control Results, Wingate, NC, USA; 17 University of Cincinnati College of Medicine, Cincinnati Children’s Hospital Medical Center, Cincinnati, OH, USA; 18 Geisinger Commonwealth School of Medicine, Danville, PA, USA; 19 Michael E. DeBakey VA Medical Center, Baylor College of Medicine, Houston, TX, USA; 20 University of Utah Health, Salt Lake City Veterans Affairs Healthcare System, Salt Lake City, UT, USA; 21 Jefferson Health, Philadelphia, PA, USA; 22 University of Pittsburgh School of Medicine, VA Pittsburgh Healthcare System, Pittsburgh, PA, USA; 23 University of Alberta, Edmonton, AB, Canada; 24 Montefiore Medical Center, Albert Einstein College of Medicine, Bronx, NY, USA; 25 University of Pittsburgh School of Medicine, University of Pittsburgh, Pittsburgh, PA, USA; 26 Perelman School of Medicine, University of Pennsylvania, Philadelphia, PA, USA; 27 University of Michigan, VA Ann Arbor Healthcare System, Ann Arbor, MI, USA

## Background

Healthcare-associated infections (HAI) are a major threat to patients across all healthcare settings. Approximately 3% of hospitalized patients in the United States (US) acquire an HAI during their stay, resulting in more than 650,000 HAIs annually.^1,2^ HAIs prolong hospital stays and increase mortality,^3^ with the estimated cost to the US healthcare system of the most common HAIs ranging from $8–$12 billion annually.^4^ HAI-causing organisms can transmit through lapses in infection prevention and can be contracted through direct contact with healthcare personnel (HCP), the hospital environment, and contaminated equipment, and to and from the patient’s own flora.^2,5–11^ The crux of preventing HAIs is an active evidence-based infection prevention program. As such, infection prevention interventions are integral to patient safety and overall healthcare operations.

Preventing and treating HAIs is a challenge. Antibiotic resistance magnifies it by increasing the complexity and cost of treatment.^12^ With rising resistance to broad-spectrum antibiotics, clinicians have come to rely on last-resort antibiotics such as carbapenems and polymyxins for the treatment of MDROs.^13^ The most common MDRO that causes HAIs is methicillin-resistant *Staphylococcus aureus* (MRSA).^14^ In recent years, increasing resistance to carbapenems has been observed in Gram-negative organisms,^15^ particularly among *Enterobacterales* (CRE),^13^ but also in other Gram-negative bacteria, such as *Acinetobacter baumannii* and *Pseudomonas aeruginosa.*^16–18^ These carbapenem-resistant organisms are a growing cause of HAIs^19–22^ and are associated with higher mortality rates than susceptible infections.^23–26^ As there are fewer drugs available to treat Gram-negative infections, and few drugs in development, the spread of carbapenem resistance has become a crisis in the US and around the world.^5,21,27,28^

A culmination of years of research in healthcare epidemiology showed that many HAIs are preventable and that effective interventions exist to mitigate the risk of HAIs for patients. Even with this progress, the landscape of healthcare epidemiology is expanding and gaps in knowledge remain. This research agenda delineates current knowledge gaps and new challenges and provides the Society for Healthcare Epidemiology of America (SHEA) Research Committee’s assessment of high-priority research topics and recommendations to advance the field of healthcare epidemiology.

### Intended use

This study sets SHEA’s research priorities over the next decade. The 2021 and 2022 SHEA Research Committees developed, based on a variety of inputs, research questions in need of “sustained, focused, and funded research” to improve patient and healthcare personnel (HCP) safety through prevention of healthcare-associated infections (HAIs) (see Methods). These research questions were posed to SHEA Research Network (SRN) participants in a survey in which respondents ranked broad topic categories and subsequently scored specific research questions on their ability to improve healthcare safety.

This research agenda is organized based on the SRN survey findings. We separated the survey’s research categories and their questions into three tiers (immediate, additional, and evolving) to highlight the items that were scored by survey respondents as highest yield in terms of potential impact to patient and HCP safety. Within the immediate priorities, we also delineated priority questions for specific settings and populations. Additional research questions were ranked from highest to lowest based on scores for potential impact on the provision of safe health care. Finally, the last tier included input from the authors on cutting-edge topics for the advancement of healthcare epidemiology and antimicrobial stewardship.

This summary provides a research agenda identified by the SHEA community at the present time; thus, the agenda is not necessarily inclusive of all research topics in the broad scope of healthcare epidemiology, infection prevention, and antimicrobial stewardship. Importantly, continued advancement and success of the fields of healthcare epidemiology, infection prevention, and antimicrobial stewardship rely on researchers’ ongoing work to reveal gaps, identify previously underappreciated or unseen challenges, and pursue novel ideas and innovative solutions that may not be outlined here. Researchers investigating questions beyond those included in this study should continue their important work.

The scope of this study does not include specific research priorities related to COVID-19 or antimicrobial stewardship, which have existing, recently published research agendas.^29,30^ SRN survey respondents were explicitly instructed to exclude antimicrobial stewardship and COVID-19 from consideration.

This study updates the SHEA research agendas: “The Evolving Landscape of Healthcare-Associated Infections: Recent Advances in Prevention and a Roadmap for Research”^31^ published in 2014, and its predecessor in 2010, “Charting the Course for the Future of Science in Healthcare Epidemiology: Results of a Survey of the Membership of SHEA.”^32^ The 2014 SHEA research agenda explored knowledge gaps and challenges in healthcare epidemiology and progress made toward addressing those issues, and built on the first SHEA research agenda, published in 2010, which called for a “national approach to HAIs” and a prioritized agenda.^32^

## Methods

The SHEA Research Network (SRN), a collaborative of healthcare facilities established by SHEA in 2012 to facilitate multicenter research projects, ranked research priority topic areas, populations, and settings identified by the 2021 Research Committee by their potential to improve patient and healthcare personnel (HCP) safety if these areas received sustained, focused, and funded research.

At the time of writing, the SRN was composed of 95 facilities located in the US and internationally. Each unique SRN facility has a point of contact responsible for responding on behalf of that facility or finding the appropriate individual(s) or departments at that facility to respond to SRN projects. SRN survey participants ranked a broad range of research topics that were submitted by members of the SHEA Research Committee or were described in past SHEA guidance documents (see Supplementary Materials, SRN Survey Results, Table 1). The survey inputs were tested by the SHEA Research Committee. The survey was distributed to 95 SRN facilities’ points of contact on October 6, November 10, and November 26 in 2021. The survey received responses from 52 of 95 SRN facilities, with 4 facilities opting out due to inability to respond at the time, for a response rate of 57% (52/91).

The survey asked respondents to select up to 5 of 14 suggested topics (see Supplementary Materials, SRN Survey Results, Table 1) and 3 of 8 populations or settings (see Supplementary Materials, SRN Survey Results, Table 3), with optional write-in entry fields, by their potential to improve patient and HCP safety if they received “sustained, focused, or funded research.” Based on their selections, respondents ranked research questions in these selected categories on a scale from 1 (least impact on improving safety if the question had well-researched answers) to 9 (greatest impact). Respondents scored specific research questions related to populations and settings from 1 to 9 by the same criteria. To get a weighted score for each research question, the mean of the scores from 1 to 9 were multiplied by the number of respondents who selected the topic or population/setting category (see Supplementary Materials, SRN Survey Results, Tables 2 and 4); where relevant, questions appeared in more than one topic category). Weighted scores were sorted from highest to lowest to develop a ranking of research questions scored as having the highest potential impact on patient and HCP safety if they were sustainably well-funded (see Supplementary Materials, SRN Survey Results, Tables 2 and 4). Each topic included a free text option for respondents to write questions not already included in survey design. As described under “Populations and Settings” section, the authors organized this section of the manuscript uniquely to address the cross-cutting nature of these patients and healthcare settings and to address potential limitations of the SRN survey in terms of the representativeness of respondents’ practice settings, roles, and specialties.

## Immediate research areas

Over 50% of SRN respondents prioritized 3 topics, with their specific research questions identified as the most urgent according to weighted scores.

### Implementation science in healthcare epidemiology and infection prevention

The three research domains identified below (also see Supplementary Materials, Suggested Methodologies, Table 1) represent priority areas with unanswered research questions in implementation science in the field of healthcare epidemiology and infection prevention:

*Priority area 1:* Identify implementation strategies and techniques that facilitate behavior change in healthcare workspaces to support the uptake and use of evidence-based infection prevention practices.

*Priority area 2:* Identify and implement strategies to support sustainability of infection prevention interventions and practices in hospitals and other healthcare settings, including those with resource constraints. This also includes developing approaches for measuring the effects of these interventions over time.

*Priority area 3*: Determine the extent to which evidence-based infection prevention practices are being implemented and sustained within and across different healthcare settings, which includes settings with differential access to resources.

Evidence-based recommendations can take 17 years to become integrated into healthcare practice.^33,34^ Implementation science seeks to overcome this delay by studying methods “to promote the systematic uptake of research findings and other evidence-based practices into routine practice, and, hence, to improve the quality and effectiveness of health services.”^35^ The focus goes beyond solution-oriented practices of quality improvement to create a generalizable knowledge base that can be utilized at a broad scale.

Implementation research often employs mixed quantitative-qualitative designs and is conducted by multidisciplinary teams that study influencing factors and behaviors at an individual and organizational level.^35,36^ Implementation science can enhance healthcare delivery.^37^ Frequently used implementation science frameworks in infection prevention^38^ and stewardship include, among others: the Consolidated Framework for Implementation Science (CFIR), the Reach, Effectiveness, Adoption, Implementation, Maintenance (RE-AIM) framework, and the Theoretical Domains framework (TDF).^38–40^

Implementation frameworks can be codified into 3 general categories: process, determinant, or evaluation.^39^ For example, CFIR helps researchers identify factors that influence the uptake of the intervention and may be particularly useful during the pre-implementation phase to mitigate operational challenges. Although the number of models and methods can be daunting, there are many resources and tools to facilitate implementation research. Among them is a dedicated implementation section in the *SHEA/IDSA/APIC Compendium of Strategies to Prevent HAIs in Acute Care Hospitals,*^40^ an implementation research primer focused on antibiotic stewardship,^39^ and resources to help with model selection,^41^ designing an implementation study,^42^ or selecting behavior change techniques.^43^ Furthermore, implementation science is the result of combining implementation research with implementation practice. Interventions and measures identified in research are not always practical to operationalize in clinical care.^44^ Involving people who are engaged in implementation practice is essential to identifying and deploying implementation strategies that are effective, sustainable, practical, and effective for complex healthcare processes.^38,45^ Implementation practice is a core function of infection prevention and antimicrobial stewardship. Bundles to decrease central line-associated bloodstream infections (CLABSIs) and behavioral approaches to antibiotic prescribing are examples of strategies found to be effective in implementation research that have been translated into practice. Implementation practice also is recommended by oversight bodies, including The Joint Commission and Centers for Medicare and Medicaid Services (CMS).^46–48^ Strategies that are flexible and multi-level, combined with interactive implementation support, may assist in evidence-based program implementation in the complex healthcare environment, including settings with limited resources.^49^

### Modes of transmission for multidrug-resistant organisms and novel pathogens

The two primary research domains below (also see Supplementary Materials, Suggested Methodologies, Table 1) have been identified to reduce uncertainty in identifying modes of transmission for multidrug-resistant organisms (MDROs) and represent priority areas with unanswered research questions:

*Priority area 1:* Increase the frequency of regular genomic sequencing and integrate results into infection prevention to improve understanding of sources of transmission events.

*Priority area 2*: Improve understanding of the role of HCP in transmission to patients directly and indirectly through the environment.

HAIs pose a significant threat to patient safety and burden to the healthcare system. Understanding transmission, colonization, and infection pathways of pathogens, and especially of MDROs, is critical to pursuing investments in future interventions.^26,50^ Existing gaps in our understanding of how MDROs spread compound the difficulty in developing and interpreting the effectiveness of interventions. With reducing costs of genomic sequencing, we can more accurately define transmission chains and thereby improve attribution around the sources and pathways of transmission.

Although infections have observable clinical manifestations, colonization is asymptomatic and can only be detected if there is surveillance. Furthermore, the time at which a patient acquires a pathogen is not observed; only the outcomes are observed days to weeks later when the patient becomes actively infected. This noisy and stochastic causal chain makes it difficult to identify the source of a colonization or infection event. Furthermore, the complexity of tracking the spread of MDROs, as resistance genes can spread rapidly between organisms of the same or different bacterial species,^13,51,52^ hampers the ability to identify transmission events and by extension their assumed routes of transmission.^53,54^

Greater investments in regular sequencing of both infections and colonization would improve understanding of sources of transmission events. In addition, improving understanding of how environmental sources become contaminated, whether directly through patients shedding bacteria or via contaminated HCP, is crucial for assessing transmission, as HCP typically have contact with both the patient and surfaces during a visit. Studies that use regular environmental sampling with sequencing can be used to link infections and environmental contamination.

In addition, defining the relative role of HCP to transmission is crucial for setting priorities for interventions. Several studies have found that HCP can transmit pathogens from patient to patient through contamination of their hands or body.^55,56^ Electronic health records (EHRs) can be mined to better understand these connections and can be leveraged for outbreak investigations.^57^ EHR data combined with sequencing of clinical isolates is capable of detecting transmissions that may otherwise have been missed. Expanding the scope of data used to generate HCP connectivity networks and matching them against hospital-based interventions will aid in understanding the drivers of transmission in the hospital, as well as the potential effectiveness of interventions. Access to whole genome sequencing of infections has broadened in recent years as the cost has come down dramatically, but still remains out of reach for many hospitals. Increased support for whole genome sequencing from the federal government, including mandates for coverage, as well as support to generalize infection control methods with common EHR systems can be a pathway to allowing hospitals with less resources to have greater access to these important methods for preventing transmission.

### Diagnostic stewardship

Five research domains identified below (also see Supplementary Materials, Suggested Methodologies, Table 1) represent priority areas with unanswered research questions in diagnostic stewardship.

- *Priority area 1:* Understand socio-behavioral, contextual, and adaptive factors that affect diagnostic test use.
- *Priority area 2:* Review opportunities for diagnostic stewardship across the continuum of testing (pre-analytic, analytic, and post-analytic stages of testing).
- *Priority area 3:* Expand diagnostic stewardship interventions beyond traditional culture-based tests to include non-culture-based tests like precursor tests, biomarkers, and molecular diagnostic panels.
- *Priority area 4*: Leverage the electronic health record (EHR) and use clinical decision support for diagnostic stewardship interventions.
- *Priority area 5*: Develop future performance metrics that align with diagnostic stewardship interventions and provide meaningful information to healthcare facilities to improve outcomes.

Diagnostic stewardship refers to optimizing the use of laboratory testing to improve patient management and treatment, with a goal of providing high-value care^58^ and improved patient outcomes. Most diagnostic stewardship interventions primarily focus on infectious diagnostic testing to promote principles of antibiotic stewardship and optimize surveillance for HAIs. These interventions traditionally have focused on outcome metrics that are publicly reported through NHSN, such as catheter-associated urinary tract infections (CAUTIs) or *Clostridioides difficile* infections. Diagnostic stewardship studies should also investigate effects on other relevant non-reportable events, such as non-device-associated urinary tract infections and catheter harm.^59,60^

Opportunities for diagnostic stewardship exist across the continuum of testing, from test ordering, specimen collection, test processing, and reporting of results,^61,62^ to the interpretation of results. Many socio-behavioral, cultural, and adaptive barriers exist at each stage of the diagnostic process. However, data related to barriers and facilitators for appropriate test utilization at each stage are limited. Specifically, implementation frameworks like the Capability-Opportunity-Motivation-Behavior (COM-B) model or CFIR could be utilized^42^ to better define these barriers.^39^ As hospitals incorporate diagnostic stewardship interventions into the EHR, unforeseen challenges will emerge. More research is needed to evaluate EHR based interventions for diagnostic stewardship and assess these interventions for unintended consequences and long-term sustainability.

There also is a need to address non-culture precursor tests like urinalysis, in which overuse can affect downstream testing, such as urine culture utilization, and lead to unnecessary antibiotic prescribing.^63^ In addition, there is increasing interest in the use of rapid molecular-based testing and biomarkers to optimize timing and choice of antimicrobial therapy in patients with sepsis.^64^ As non-culture-based tests become popular, we need to expand diagnostic stewardship beyond traditional culture-based tests to include multiplexed molecular diagnostic panels,^65^ metagenomic next-generation sequencing testing, and biomarkers. These tests present numerous opportunities and challenges that differ from traditional culture-based tests.^66^ Studies are needed that demonstrate the cost-effectiveness, long-term outcomes, and sustainability of diagnostic stewardship interventions related to non-culture-based testing.

Diagnostic stewardship can be leveraged to de-implement testing practices that increase healthcare inequities.^67^ There are several examples of racial and ethnic disparities as it relates to overuse of healthcare services. For example, Black and Hispanic patients with dementia were more likely to receive inappropriate feeding tube use as compared to White patients. Similarly, Black and Hispanic patients were more likely to receive unnecessary cardiac screening and preoperative testing.^68^ Furthermore, overprescribing or overuse leads to fewer resources for patients who are most in need of these services.

To improve patient outcomes, performance metrics should reflect infectious and noninfectious complications and align with quality improvement efforts.^59,69^ For example, hospitals are currently incentivized to concentrate their efforts on decreasing NHSN CAUTI events instead of urinary catheter harm or treatment of asymptomatic bacteriuria. To improve patient care, metrics should align with diagnostic stewardship interventions and provide meaningful information back to facilities.

## Populations and settings

SRN respondents ranked eight populations and settings according to the need for them to receive sustained, focused, and funded research (see Supplementary Materials, SRN Survey Results, Table 3). The authors organized this section of the manuscript uniquely to address research needs for these populations and settings.

- The authors incorporated research priorities for acute care hospitals, ambulatory/outpatient care, and home health care within relevant areas of the manuscript’s immediate and additional research priority sections. These settings are not addressed separately in this section of the document.
- Research priorities for patients who are among minority groups, are under-represented, and/or are socioeconomically disadvantaged, living in resource-limited settings, and/or living with immunocompromising conditions are handled similarly. These research priorities are threaded throughout the manuscript’s immediate and additional sections and are not addressed separately here.

This section addresses research needs for:

- Post-acute care settings and nursing homes
- Pediatric patients and settings

Of note, the pediatrics discussion includes topics beyond those addressed in the SRN survey to address the potential limitation that pediatric facilities were under-represented among the recipients and respondents to the SRN survey.

### Post-acute care and nursing homes

Several areas in post-acute care and nursing home infection prevention were identified as high priority for investigation (also see Supplementary Materials, Suggested Methodologies, Table 5):

- Develop practical interventions to reduce common HAIs and transmission of MDROs, which can be implemented and sustained in accordance with available resources in post-acute and nursing home settings.
- Conduct well-designed epidemiologic studies to evaluate the role of environmental contamination in transmission of viral and bacterial pathogens in nursing homes.
- Develop and test individual, facility-level and systemic strategies to support infection prevention training and practices in post-acute and nursing home settings, including developing models for collaboration with local and national entities to promote rapid responses to infectious outbreaks and pandemics.

#### Develop practical interventions to reduce common healthcare-associated infections and transmission of MDROs

Infection prevention in post-acute care facilities and in nursing homes is challenging. More than 153,000 nursing home residents have died of COVID-19 since the pandemic,^70^ constituting 12% of nursing home residents in the US.^71^ Pervasive staff and leadership turnover affects overall care, including infections, patient satisfaction, and quality-of-care.^72–75^ Recent studies show that support from external entities such as hospital-based infection prevention teams or local and state health departments can enhance infection prevention in nursing homes.^76,77^ These collaborations also provide opportunities to conduct multi-site clinical research. Prior successful examples include a multi-state national implementation project to prevent CAUTI in nursing home residents,^78^ and implementation of directly observed hand hygiene by hand hygiene ambassadors.^79^

MDRO infections are common, difficult to treat, and remain a major concern within the post-acute and nursing home setting. The estimated rate of MDRO infections among nursing home residents across the US is 4.2%, ranging from 1.9% to 11.4% in individual states, based on the CMS Long Term Care Minimum Data Set.^80^ Over half of all nursing home residents are colonized with MDROs, as illustrated in a study involving 1,400 residents in 28 nursing homes.^81^

#### Conduct well-designed epidemiologic studies to evaluate the role of environmental contamination in transmission of viral and bacterial pathogens in nursing homes

Environmental contamination with MDROs is prevalent and persistent. Findings from a cluster randomized trial suggest that implementation of multicomponent interventions, including enhanced barrier precautions, chlorhexidine bathing, MDRO surveillance, environmental cleaning, hand hygiene promotion, and HCP education and feedback can reduce the odds of MDRO prevalence in patients’ environment by 43%.^82^ Hence, future studies that build on these findings are needed urgently. These studies should use a variety of research methods, including cluster or stepped-wedge study designs and implementation science to design practical, sustainable, and cost-effective interventions with the explicit goal of reducing clinical infections due to MDROs while maintaining residents’ dignity, recreational activities, and independence.

#### Develop and test individual, facility-level and systemic strategies to support infection prevention training and practices in post-acute and nursing home settings, including models for collaboration with local and national entities

CMS requires that nursing homes have robust infection prevention programs. Yet, infection prevention training for nursing home personnel often remains underdeveloped. A cross-sectional survey of 2,514 randomly sampled nursing homes on infection preventionist training, staff turnover, and infection prevention program characteristics, found that less than 3% of infection preventionists were certified in infection prevention.^83^ Training and education of nursing home staff is integral to reducing infection risk and antimicrobial overuse in nursing homes. For example, the non-evidence-based practice of excess urine culture ordering in catheterized patients leads to antibiotic exposure and therefore risk of infection with MDROs.^84^ Further investigation into educational interventions to empower nursing home residents, which can be individually tailored, interactive, or structured also will benefit nursing homes’ safety.^85^

Furthermore, it’s crucial to recognize that certain populations, particularly racial and ethnic minorities, may face disproportionate impacts in post-acute care and nursing home settings due to systemic healthcare access and quality disparities. Under-resourced settings with limited staffing and resources also encounter unique challenges in infection prevention. Recent studies indicate higher COVID-19 cases and deaths in nursing homes with more racial/minority residents, as well as elevated incidence rates in low socioeconomic neighborhoods compared to high socioeconomic neighborhoods.^86–88^

Additional studies reveal distinct risk factors for COVID-19 infection and mortality. Densely populated counties have a higher infection risk, while areas with higher disability and poverty rates experience increased death rates.^88^ Early studies emphasize the need for systemic solutions. For example, a study on reducing racial disparities in influenza vaccine uptake found that implementing strategies like standing orders, verbal consent, and routine review of facility-wide vaccination rates in nursing homes significantly reduced racial vaccine gaps.^88^

Future research should prioritize effective targeted strategies encompassing individual, facility-level, and system-level approaches to reduce healthcare disparities.

### Pediatrics

Several areas in pediatric infection prevention were identified as high priority for investigation (also see Supplementary Materials, Suggested Methodologies, Table 5):

- Effective and sustainable practices to decrease pediatric HAIs, including in rural and resource-limited settings
- Effect of visitation policies on in-hospital transmission and other important clinical and service outcomes
- Prevalence of non-device-associated HAIs in children
- Reliable HAI definitions, especially for the neonatal population
- Effective and sustainable practices to decrease pediatric HAIs

Future research should apply quantitative and qualitative methodologies to identify modifiable factors associated with adverse pediatric events. Understanding how system factors may mitigate HAIs and affect long-term improvement involves developing reformative and sustainable care models through broad examination of work environments and consideration for systematic application of implementation frameworks to study and evaluate processes of highly specialized healthcare teams.^89^ Recent studies introduce the critical importance of diagnostic stewardship to mitigate HAIs and reduce broad-spectrum antibiotic use, but gaps remain regarding how to maintain improvements longitudinally to prevent pediatric HAIs.^90^

As pediatric care becomes progressively complex, healthcare facilities have identified an increased risk of device-associated infections, including CLABSIs and CAUTIs,^91^ in pediatric patients. Collaborative efforts spanning single and multicenter facilities reported sustained reduction of CLABSI and CAUTI events when they focused on evidence-based, bundled care practices.^89,92^ Children also can undergo invasive procedures such as ventriculoperitoneal shunt placement, spinal hardware placement, or congenital heart disease repair, which can have unique factors that contribute to risk for device-associated surgical site infections.

We encourage investigators to explore multi-faceted implementation strategies to support sustained improvements in pediatrics. Effective and sustainable practices rarely reside in isolation. Integrated approaches to address influences from diagnosis to treatment, including factors that influence decision-making for testing, could be investigated through observational and quasi-experimental designs, implementation designs, and mixed methods evaluations. We encourage research into implementation designs that demonstrate the impact on patient safety. As identified with the COVID-19 pandemic where resource allocation (e.g., infrastructure, people, supplies) was significantly affected, system-level local and organizational contextual factors that influence the long-term safe management of high-risk devices warrant better understanding.

#### Impact of visiting policies and related infection prevention measures on in-hospital transmission of viral illnesses in pediatric and neonatal settings

The benefits of family presence at bedside for hospitalized neonates, infants, and children is well-established, though visitors pose a potential risk of exposing hospitalized patients to transmissible viral illnesses.^93^ A limited body of literature supports the potential benefits of visitor screening and seasonal visitor restriction policies in the pediatric acute care setting, for reducing the spread of respiratory viral illness, including during the pre-pandemic period.^93–96^ Future studies using methodologically robust designs (e.g., quasi-experimental studies, randomized controlled trials) should be performed to more fully evaluate these findings, explore the benefits and harms of year-round versus seasonal policies, and investigate optimal approaches to minimize transmission risk from visitors (including rigorous evaluations of interventions to mitigate risk, such as masking and visitor hand hygiene). In addition, studies evaluating the effectiveness of visitor screening and/or restriction in the pediatric post-acute care and long-term care settings are needed. Along with assessments of clinical effectiveness, we also encourage research in the assessment of harms (e.g., reduction in visits for children, perceptions of healthcare quality and experience, health communications), including qualitative investigations that include pediatric patients and their family members and consideration of contextual factors (e.g., clinical setting, patient severity of illness). Finally, we encourage the integration of assessments of benefits and harms of infection prevention strategies using a variety of research designs, including economic evaluations.

#### Prevalence of non-device-associated HAIs in children (including respiratory viral infections)

Respiratory viral transmission in healthcare settings is a complex interplay of the host, contacts, the environment, and the pathogen. Research is needed to advance our understanding of the frequency of respiratory viral transmission in pediatric healthcare settings, risk factors for respiratory viral transmission in pediatric settings, including evaluations of disparities in both transmission risk and clinical outcomes, and effective interventions for interrupting spread that considers these factors. Attention should be paid to how access to resources to mitigate transmission risk impacts outcomes. Studies should assess directionality of spread (e.g., from HCP to pediatric patients, or pediatric patients to HCP) and ideally include viral genomics data, rather than purely epidemiologic linkages to establish transmission patterns. We encourage researchers to estimate the effectiveness of different infection prevention strategies for reducing healthcare-associated transmission (e.g., randomized controlled trials and quasi-experimental studies) and consideration of setting (e.g., inpatient versus outpatient settings of care, urban versus rural hospital settings) and pathogen, as impact of interventions designed to interrupt transmission may vary according to contextual factors. We also encourage investigations into the prevalence of asymptomatic respiratory viral infections and the role that asymptomatic infections in pediatric patients play in transmission.

#### Definitions of HAIs among the neonatal population

HAIs account for a significant proportion of morbidity and mortality in neonates and can lead to neurodevelopmental impairment among survivors; however, there are substantial knowledge gaps in how HAIs should be diagnosed and managed in this population.^97,98^ Positive blood culture is the gold standard for diagnosis, but the limitations of this diagnostic approach include the delay between collection and positivity, false positive results from contamination, and submission of inadequate blood volume, which can be particularly challenging in extremely preterm infants.^99–101^ The concept of “time-to-positivity” to diagnose CLABSI^102^ has not been well studied in the neonatal population, likely because of the relatively small-sized lumen of their central venous catheters. Future studies should focus on the optimal biomarkers and/or molecular tests to allow early diagnosis of neonatal bloodstream infections.

Neonatal ventilator-associated pneumonia (VAP) remains a leading cause of antibiotic use in the NICU.^103–106^ Barriers to improvement in neonatal VAP practices include the lack of standardization or guidelines for definition and surveillance, and the inability to differentiate VAP from other non-infective causes of respiratory deterioration. This results in major practice variations as well as antimicrobial overuse.^107^ To evaluate the treatment outcomes and impact of quality improvement efforts properly, we urge the development of specific standardized diagnostic criteria for VAP in term and preterm neonates.

Urinary tract infection (UTI) can result in renal scarring and long-term consequences,^108^ but current practice guidance on UTI diagnosis and management only targets patients down to 2 months of age.^109^ Specifically, there is a lack of data to support the most appropriate cut-off value for colony-forming units to determine UTI in term and preterm infants. The lack of a consistent definition of UTI in this population prevents us from understanding the magnitude of the problem and how to improve practice. We encourage the development of clinical and epidemiologic studies to find evidence-informed strategies for interpreting microbiologic culture results and developing treatment recommendations.

## Additional research areas

Nearly 40% of SRN respondents identified the following topics, populations, and settings, listed in descending order by the research questions’ weighted scores (see Supplementary Materials, SRN Survey Results, Table 2).

### Infection prevention during public health emergencies

The three research domains identified below (also see Supplementary Materials, Suggested Methodologies, Table 2) represent priority areas with unanswered research questions in infection prevention in public health emergencies:

- *Priority area 1:* Identify how hospitals can combat HAIs due to MDROs during public health emergencies.
- *Priority area 2:* Determine how constrained staffing or other resources affect the implementation and sustainability of existing and new HAI prevention strategies.
- *Priority area 3:* Understand the impact of public health emergencies on the epidemiology of HAIs.

Recent studies reported an increase in HAIs during the COVID-19 pandemic,^110,111^ likely due in part to diverted attention from routine infection prevention and antimicrobial stewardship efforts during the emergency. Infectious disease events related to emerging pathogen outbreaks, bioterrorism, or mass casualties often occur unexpectedly and cause significant morbidity, mortality, and economic disruption. These events require preparedness at global, national, regional, and facility levels.^112^ Healthcare systems are challenged with limited guidance on prioritization of personal protective equipment (PPE), isolation rooms, considerations for discharging potentially infectious patients to post-acute facilities, practices related to care of immunocompromised patients and other vulnerable populations, and more. Healthcare systems in low- and middle-income countries are particularly challenged for resources, which was evident during the recent COVID-19 pandemic.

Before public health emergencies occur, Brookes at al emphasize that three levels of scanning are necessary to assist with planning and anticipating disease occurrences—the environment for news, the horizon for occurrence close to healthcare facilities, and when the emergency occurs in the hospital, facility surveillance and risk assessment.^113^ Randomized controlled trials are not suited to providing actionable information in public health emergencies. In their absence, we encourage well-designed qualitative studies, surveys, and innovative study designs that borrow from disciplines outside of medicine such as human factors engineering, sociology, behavioral sciences, and related fields.

Best practices for engaging HCP and leading change during a crisis need to be identified through systematic qualitative research. A qualitative study evaluated key factors driving hospitals’ performance in biological disasters.^114^ Another key article, which could potentially be applicable to infection preventionists, found that four domains of competencies are most important for nurses involved in disaster response: critical thinking, assessment, technical skills, and communication skills.^115^ Research using human factors engineering may be warranted to improve adherence to hand hygiene, PPE, and catheter maintenance, particularly during public health emergencies.^116^ The need for disaster-specific infection prevention teams warrants further research as well.^117^ As part of preparedness, effective strategies are needed to train frontline HCP in diverse settings, including those in resource-limited parts of the world, rapidly and on a large scale, as well as approaches for communicating epidemiology-based shifting guidance on an ongoing basis. Qualitative studies to explore potential for resource-sharing across facilities during disasters are needed. Infection prevention workforce needs during infection-related public health emergencies must be studied in high-income, as well as low- and middle-income countries, to provide guidance to health systems and public health agencies to effectively respond to the public health emergency as well as continue routine surveillance and prevention activities related to HAIs and MDROs. Finally, we need to determine best practices for sustainability and scalability of infection prevention across facilities in advance of the next crisis.

### How to use data to prevent HAIs

The three research domains identified below (also see Supplementary Materials, Suggested Methodologies, Table 2) represent priority areas with unanswered research questions in how to use data to prevent HAIs:

- *Priority area 1:* Identify optimal study designs and statistical methods for data-driven healthcare epidemiology.
- *Priority area 2:* Determine which risk assessment models can be used to predict patient harm.
- *Priority area 3*: Develop strategies for using data and analyses to consistently provide clinically significant and actionable information to clinicians.

High-quality data is of prime importance to healthcare epidemiology. Data on infection rates informs patient care, identifies sources of transmission and high-risk areas of health care, provides vital information for assessments, and monitors intervention effectiveness. This same information is repurposed for operational, financial, and reputational needs of both individual units and hospitals.^118–120^ Underlying all uses is the need for metrics that are both valid (in that they correctly measure what they claim) and dependable (in that they support empirically informed decisions).

Methodologically, two broad challenges present themselves to data-driven decision-making in hospital epidemiology. The first is the ability to collect data and link it directly to patient outcomes. This is outlined in greater detail in the preceding section on transmission. Readily available data gleaned from clinical cases may provide an incomplete picture because the causal pathway that leads to an infection is long and stochastic. The second challenge is the development of risk assessment models to guide intervention *prior* to patient harm. In general, to-date, risk prediction models in medicine have not lived up to their potential^121–124^ likely due to the small samples and noisy causal pathways that characterize HAIs.^125^ One example is the challenge of balancing prediction with the use of variables that may be manipulated. In addition, mathematical models are limited by the accuracy of inputs and effect estimates. We need a close link between modeling and observational studies to realize the full potential of both.^126,127^ These challenges affect researchers and clinicians who implement interventions in their own settings. Randomized trials are often difficult to conduct in hospital epidemiology for ethical and practical reasons, though new trial^128^ and non-trial designs^129^ can overcome some of these problems.

Central priorities include that data analyses may provide clinically meaningful, actionable, and sustainable information. For example, risk prediction models that guide interventions must be continuously updated to ensure they remain useful. Although these challenges are present everywhere, they are especially present in low-resource settings, including rural communities and low and middle-income countries. The COVID-19 pandemic has also highlighted the need for local-level expertise during public health emergencies. Additionally, concerted effort must be made to ensure that the data collected are as unbiased as possible across a broad spectrum of variables—race, sex and gender, body size and socioeconomic status to name but a few that healthcare data has often struggled with. The most advanced algorithms available cannot overcome bias that is rooted in the data itself.

The continued threat of HAIs requires answers to these methodological and practical challenges. Infection prevention teams should lead this effort, taking an active role in assuring data are well-collected and unbiased. The tools and techniques of data analysis must generate valid and reliable metrics that are actionable and sustainable at an institutional level on which facilities and practitioners can make decisions. Finally, infection prevention teams must determine methods to communicate the outcomes of these efforts to those who can implement them to improve patient outcomes.

### Role of the environment

The three research domains identified below (also see Supplementary Materials, Suggested Methodologies, Table 2) represent priority areas with unanswered research questions about the role of environment in colonization of patients and HCP:

- *Priority area 1:* Quantify the risk of acquiring pathogens from the healthcare environment, including risk of infection.
- *Priority area 2*: Identify reservoirs within the healthcare environment that are most important for to pathogen transmission to patients.
- *Priority area 3:* Determine strategies to improve quality of cleaning and disinfection through application of existing technologies, new methods, and novel approaches.

The healthcare environment, including surfaces and equipment, is highly contaminated with pathogens.^130,131^ Despite routine cleaning, studies have demonstrated persistent contamination of surfaces with MDROs and have identified associations between contamination and risk of infection.^132–137^ However, several gaps persist in our understanding of how patients become infected with pathogens from the healthcare environment and how to prevent transmission.

Although studies have repeatedly demonstrated that contamination of the healthcare environment occurs, its relative contribution to HAIs is unknown. The risk of infection has been associated with prior room occupants, potentially due to inadequate terminal room cleaning;^138^ however, studies attempting to demonstrate this risk directly are limited.^139^ Additionally, cohort studies have shown that positive environmental cultures predict contamination of HCP,^140,141^ supporting the idea that environmental contamination can result in transmission of pathogens to adjacent patients. Further research is required to quantify the risk of transmission with subsequent HAIs to better define quantitative, intermediate goals.

Our understanding is still evolving about the reservoirs that harbor epidemiologically important pathogens in healthcare environments. Although high-touch surfaces and hand or glove contamination are known vehicles, other sources of pathogens require identification and quantification of risk to patients. After understanding where pathogens reside in the healthcare environment, we can better target and refine mitigation strategies. Potential reservoirs in the healthcare environment that have not yet been fully evaluated may include reusable, mobile, or shared medical equipment^142^ and shared spaces.^143^ Healthcare water sources that develop biofilms (e.g., sinks, premise plumbing, ice machines, and/or heating/cooling, and perfusion machines) are particularly important for future investigation, as water-biofilm sources have been responsible for facility and global outbreaks.^144–147^ Further, we do not know the role of wastewater surveillance in detecting immediate risks of HAI for patients and when to trigger mitigation strategies.^147,148^

Environmental cleaning is a complex, multistep, mostly manual process with many opportunities for human error.^149^ Research is needed to identify practical methods to improve quality and consistency of environmental cleaning. Although there has been an increase in utilization of touch-free disinfectant technologies in the past decade, these have not substituted the need for effective manual cleaning.^150–153^ To fill these knowledge gaps, we need investigation into the clinical significance of surface contamination prior to terminal room cleaning and how to prevent patient contamination of surfaces, including the role of continuous disinfectant technologies. Research is also needed to determine the specific benefits of enhanced cleaning and disinfection in immunocompromised patient populations. All patients deserve to be cared for in safe, clean, healthcare facilities. Inequity in healthcare access and resources likely impacts the safety of healthcare environments; however, the degree to which inequities affect healthcare environment cleanliness has not been well-defined or addressed.

Disinfection of persistent reservoirs in sinks, drains, and other water sites remains challenging due to biofilm formation.^133,145,154,155^ Additional research is needed to identify strategies to effectively disinfect these reservoirs and to understand the potential benefit of removing or relocating such sites further from patient care areas. Finally, as new pathogens emerge, research must again assess optimal cleaning strategies for the healthcare environment based on pathogen-specific characteristics.^143^

### Healthcare personnel wellness and burnout

The three research domains identified below (also see Supplementary Materials, Suggested Methodologies, Table 3) represent priority areas with unanswered research questions in healthcare personnel wellness and burnout:

- *Priority area 1:* Create and establish methods to monitor and maintain the sustainability of the healthcare workforce.
- *Priority area 2:* Investigate methods to promote healthcare epidemiology personnel workforce resiliency.
- *Priority area 3:* Identify and implement effective communications strategies to support HCP.

“Burnout” is defined by the US Department of Health and Human Services as “an occupational syndrome characterized by a high degree of emotional exhaustion and depersonalization (i.e., cynicism), and a low sense of personal accomplishment at work.”^156^ Drivers of HCP burnout include societal, organizational, structural, and cultural factors, with specific examples including excessive workloads,^157^ administrative burdens, and lack of support from leadership and colleagues.^156^ Burnout is contributing to HCP contemplating leaving the medical profession entirely, which would exacerbate a pre-existing shortage,^158^ directly affecting healthcare workforce sustainability.^159^ Difficulties achieving desired work-life balance, which may impact different demographic groups in different ways, may also contribute to healthcare workforce shortages, exacerbating challenges.

Infection prevention and control departments are frequently understaffed and under-resourced, even during non-crisis times.^160–164^ There is no slack in the system when a crisis emerges that increases the overall workload. Addressing these problems requires innovation in how staffing needs and full-time equivalents (FTEs) are calculated and distributed. Future research should evaluate how staffing and other resource availability affect existing and new HAI prevention strategies (see Supplementary Materials, Suggested Methodologies, Table 3). Specifically, reliable tools for measuring workforce capacity and resilience would help quantify resource availability and identify thresholds required to maintain high-quality, day-to-day infection prevention staffing regardless of the external context and competing priorities. These challenges may be particularly acute in small and non-academic institutions, which may have less access to resources to address workforce capacity gaps. Rather than a “one-size-fits-all” approach, researchers should consider developing and testing tailored strategies for different facility types (e.g., small versus large hospitals, urban versus rural, academic affiliation status versus non). Researchers may also consider investigating flexible work strategies to support members of the healthcare workforce who might not otherwise be willing or able to participate in IPC activities.

Identification of novel reimbursement and staffing strategies that allow for slack in the system such that HCP are available during unanticipated emergencies to absorb additional tasks during those situations are critical for addressing sustainability and resiliency.^165^ Potential strategies that could be investigated through experimental, quasi-experimental or implementation designs include training medical staff in some aspects of infection prevention to expand the workforce, creating novel partnerships between small and large hospitals, telemedicine strategies, and hybrid staffing models (e.g., part-time FTE in IP that is already trained and can be re-allocated during an emergency).^166^

Healthcare economics investigations that systematically evaluate the “value-add” of different tasks to develop a rational and evidence-based hierarchy of IPC tasks and interventions is also a priority. Such research could lead to the creation of evidence-informed “tiered approaches” to infection prevention activities (e.g., rating day-to-day activities as “essential” versus “high priority” versus “optional/lower priority” activities). Highlighting essential versus optional activities may help maintain quality standards when available staff cannot support all infection control activities. As suggested by SHEA survey respondents, researchers are called to develop validated measurement tools and scales that are dependable, reproducible, and equitable. In line with the principles of Open-Source Science,^167^ to reduce overall workload burden and reduce redundancy, we need innovative and equitable mechanisms for resource-sharing and distribution of tasks across healthcare networks and systems, and the creation of large repositories of infection prevention toolkits that can be shared and then locally adapted to reduce repetitive work and overall workload, along with IT strategies including EHR systems for truly automated surveillance to reduce workload from automatable activities.^168–170^

Innovations in the field of health communications, including advancements in understanding effective communication strategies, how communications strategies can to be tailored to be culturally appropriate, sensitive, and equitable, and how information and misinformation is disseminated and diffused are needed to improve the adoption of best infection prevention practices and to reduce HCP’s perceptions that their efforts are futile or their impact on improving healthcare quality is minimal.

### Device-associated infections

The three research domains identified below (also see Supplementary Materials, Suggested Methodologies, Table 3) represent priority areas with unanswered research questions in device-associated infections:

- *Priority area 1*: Improve infection surveillance definitions and goals.
- *Priority area 2*: Develop novel device technologies or methods to minimize the incidence of infection.
- *Priority area 3*: Determine how best to prevent device-associated infections in outpatient and high-risk populations.

Medical devices play a vital role in health care, but also carry a risk of infection. Key device-associated infections are monitored by nationally reported surveillance programs that are tied to hospital rankings and pay for performance. Healthcare epidemiology has opportunities for improvement in the approach to surveillance, the development of new device technologies, and strategies to reduce infections in special and under-resourced populations.^171–176^ Studies should determine the presence and magnitude of outcome disparities among racial, ethnic, or other social groups. Interventions should be developed to lessen the disparities.

Healthcare epidemiology requires an evidence-based method to determine the lowest achievable frequency of HAIs. A better understanding of the lowest achievable infection rates would guide efficient use of infection prevention resources toward truly preventable events.^171–175^ Furthermore, identification of infection prevention interventions that improve patient clinical outcomes, beyond infection rates, would highlight interventions that carry greatest benefit for patients.

Infection prevention programs need to know the benefits and risks of novel device technologies and approaches to device maintenance, including devices’ features for colonization or biofilm-resistance and mechanical or biological ways to reduce bacterial burden.^177^ Studies of devices and technologies should be powered to include patient-focused outcomes to define when invasive devices (e.g., mechanical ventilation) should be preferred to less invasive devices (e.g., non-invasive positive pressure ventilation), and vice versa, as outcomes focused only on microbial contamination can overestimate infection rates. Studies should include appropriate patient selection to maximize benefit over risk and should include a standard of care comparator. Evaluation of novel device technologies and maintenance, especially those that include antimicrobials or disinfectants, should report unintended effects, such as acquired antibiotic-resistance, device degradation, or device dysfunction (e.g., catheter occlusion or impaired ventilation).^178^ Technologies that employ non-invasive strategies (e.g., non-invasive ventilation, external catheters) should continue to be a focus of investigation.^179,180^

Several patient populations are at unique risk for developing device-associated infections. Patients who have prolonged device utilization, mostly in the outpatient or long-term care setting, may not benefit from traditional inpatient strategies for infection prevention. Research is needed to further define the epidemiology of device-associated infections and tailor prevention strategies in these groups, including outpatient dialysis and patients on home or clinic-based infusion therapy.^166,181–183^ Additionally, patients receiving treatment for cancer, particularly hematologic malignancy, frequently develop infection due to severe immunocompromise and associated skin and mucosal barrier injury.^184^ Cohort studies can better define the incidence of infection in high-risk outpatient populations, and interventional trials can identify effective infection prevention strategies. Such patients may have frequent and prolonged exposure to healthcare settings, resulting in acquisition of resistant pathogens.^185^ Finally, infection prevention practices should be studied for devices that lack effective strategies and are responsible for significant morbidity and antibiotic exposure, such as long-term ventricular assist devices. Opportunities for research include not only defining opportunities to predict and prevent device-associated infections, but also to reduce infections occurring from MDROs.

### The role of the community in the spread of infections in health care

The three research domains identified below (also see Supplementary Materials, Suggested Methodologies, Table 3) represent priority areas with unanswered research questions related to the role of the community in the spread of infections in health care:

- *Priority area 1*: Describe the epidemiology of HAIs in community settings, including higher risk patients and care settings.
- *Priority area 2*: Develop and test effective strategies for community-onset HAI surveillance and infection prevention in non-hospital settings.
- *Priority area 3*: Define barriers and challenges to infection prevention in community settings and develop and test effective mitigation strategies.

Data suggest that the community prevalence of MDRO colonization and infection is rising—both among those who have underlying health conditions, and those who do not.^186,187^ Almost half of patients with extended-spectrum beta-lactamase (ESBL)-producing *Enterobacterales* infections do not have histories of prior hospitalization or recent invasive procedures. Only forty percent of community-associated *C. difficile* cases had exposure to outpatient healthcare services.^188,189^

Several factors have contributed to the rise in CO-HAIs. Advances in the medical management of complex health conditions and the steady growth of an elderly population with multiple comorbid conditions led to increasingly complex care being delivered in the home and other non-acute care settings.^190,191^ The COVID-19 pandemic accelerated this shift when hospitals began “hospital-at-home” programs, clinics transitioned visits to telemedicine, and nursing homes/long-term care facilities handled an influx of patients within an already overloaded system;^192^ yet the prevalence and burden of community-onset CLABSIs, surgical site infections (SSIs), and CAUTIs is largely unknown.

There are several challenges to evaluating CO-HAIs. Community health care has been defined as medical services provided to individuals who are not admitted to inpatient hospitals. Community healthcare settings are highly diverse, spanning nursing homes and long-term acute care hospitals, outpatient clinics (e.g., surgical and infusion centers), community housing (e.g., group homes), hemodialysis centers, allied healthcare settings (e.g., dental clinics, physical therapy/rehabilitation), and home healthcare agencies. Different settings carry varying degrees of risk and cannot be assumed to have the same HAI definition. For example, National Healthcare Safety Network (NHSN)-defined dialysis events use a different definition as NHSN-defined acute care CLABSIs.^193^ Other than for hemodialysis centers, there are gaps in standardized surveillance definitions and accurate denominators for CO-HAIs for community healthcare settings.^194^ Inequity in access to care can further impact representation of under-resourced or disadvantaged communities in surveillance efforts. Furthermore, cohesive surveillance systems to capture reports of CO-HAIs do not exist and attempts to perform surveillance have relied on incomplete data sets.^195,196^ Development and validation of standardized surveillance definitions for CO-HAIs is needed.

Furthermore, infection prevention and antibiotic stewardship expertise in community settings is severely limited. Outpatient outbreaks have been associated with lapses in infection prevention practices.^197^ Current infection prevention and antibiotic stewardship efforts have focused mostly on acute care settings, and their successes have yet to be translated to the prevention of CO-HAIs. Community healthcare settings vary significantly in the patient population and medical services that are delivered, which can affect the type of infection prevention strategies needed, along with differences in funding, structure, and resources. Research addressing the specific needs of under-resourced and socioeconomically disadvantaged communities is a high priority to assure pragmatic and high-value strategies can be developed to improve infection prevention in these populations. In addition to the need to translate acute care infection prevention strategies to community settings, effective solutions to reduce HAIs in outpatient settings require public health agency support in investment.

Finally, the COVID-19 pandemic showed the interconnected nature of communities and how schools, community organizations, colleges, and businesses affect public health. Workforce development and encouragement for students to study the health sciences is one way to enhance the overall health of the community, facilitate a community’s public health responsiveness to outbreaks, improve uptake of interventions, and reduce misinformation.^198^

Communities also are interdependent on the global scale. Policies, politics, and priorities in one country directly affect populations in other countries. *There can be no global economy without global public health*. Researchers must creatively propose—and funders should support—research initiatives that allow us to develop infection prevention infrastructures across countries and populations.

### The role of social interaction and engagement for patients

The four research domains identified below (also see Supplementary Materials, Suggested Methogologies, Table 4 ) represent priority areas with unanswered research questions in the role of social interactions and engagement for patients:

- *Priority area 1:* Clarify the benefits and costs of different types of isolation interventions.
- *Priority area 2*: Understand the appropriate implementation of visitor restrictions.
- *Priority area 3:* Determine the role patients have in preventing infections associated with health care.
- *Priority area 4:* Demonstrate the effects of using best practices to communicate to the public about HAIs.

Visitors to healthcare settings may carry infection or become infected, prompting restriction between the public and patients.^94,199–201^ The balance of costs and benefits of these approaches are difficult to quantify, and communication that promotes behavior change is complex. Better understanding is needed regarding effective communications methods with the public and patients. The challenge of communication and interaction are amplified by inequitable access to care and information as well as cultural and language differences and barriers.

Transmission-based precautions prevent some infections, but we need to better understand the potential for psychological, environmental, and financial consequences of these practices.^202–204^ Some studies have shown unintentional effects of transmission-based precautions on patients, along with barriers to providing care.^205,206^ Individuals who reside in long-term care facilities can be particularly affected by universal or expanded use of transmission-based precautions.^207^ Increases in the use of PPE associated with some transmission-based precautions can lead to waste with downstream environmental consequences.^202^ Masks, disposable gowns, and gloves are sources of microplastics^208^. PPE use may contribute to financial challenges for healthcare facilities. Facilities that serve marginalized populations are affected disproportionately.^204^ Knowledge gaps remain around how to reprocess safely and recycle to reduce medical waste.^203^

We also need further research into the effect of visitor restriction policies on HAI reduction, which has not been consistently studied or correlated.^209,210^ Complexities include practice variations, the effect of patients’ resumption of close contact with others, and the effect of the community on patient transmission versus the important emotional, psychosocial, and physical support visitors can provide to patients, especially in settings such as the neonatal intensive care units (NICUs).^209,211–213^ Except for novel infectious agents, many pathogens already are present in the community due to patients’ and visitors’ movement in and out of health care. Policies for visitors should be evaluated within the context of age, comorbidities, socioeconomic considerations such as restrictive visitation hours and ability to access technological alternatives to in-person visitation, and the cost-benefit of visits with accompanying children.^201^

Studies are needed to understand how engagement may change according to context and situation. There are several ways that patients can influence the effectiveness of IPC ranging from involvement in self-care (e.g., proper hand hygiene, catheter management) to patients and families’ contributions to monitoring staff practices (e.g., observing hand hygiene) and speaking up. Conversely, concerns may include variability in patients’ knowledge, ability, and willingness; the potential for loss of trust in healthcare staff, and the ethics of shifting monitoring burden to patients and caregivers.^214,215^ There are few systematic reviews about the effectiveness of patient involvement across infection-related topic areas.^216,217^

As described by the National Quality Forum (NQF), patient-reported outcomes (PROs) are defined as “any report of the status of a patient’s (or person’s) health condition, health behavior, or experience with health care that comes directly from the patient, without interpretation of the patient’s response by a clinician or anyone else.”^218^ Commonly reported domains include health-related quality of life (including functional status); symptoms and symptom burden (e.g., pain, fatigue); experience with care; and health behaviors (e.g., smoking, diet, exercise). A recent report identifies best practices for selection and data collection of PRO measures; however, challenges remain related to reliable, efficient data collection and concerns about the accuracy of the information. For example, a systematic review of PROs for community-acquired pneumonia found that none of the five validated community-acquired pneumonia (CAP)-specific instruments were supported by high-quality evidence of their content validity.^219^

Further study is needed into whether publicly reported metrics communicate a useful message, and how patients and caregivers make decisions using these metrics. Factors that affect the usefulness of publicly reported quality metrics include topics; interest and relevance; accessibility; consumer health and education level; report card design and display; complexity and interpretability of metrics; and availability of choice under insurance plans.^220,221^ Early studies suggest report cards were not widely used and did not change consumer or HCP behavior.^222,223^ Little is known about the extent to which report card information is useful to individuals with limited English proficiency and/or access to online resources. Though several HAI-related measures are reported on government websites like Care Compare, more research is needed to determine if the information is meeting the stated goal of helping consumers make informed decisions about where to get their health care.^224^

Although extensive scholarship exists on the topic of communication, which is essential to bring about change in public health and health care, little directly addresses HAI prevention.^225^ Patients, visitors, HCP, decision-makers, and the public require different approaches.^225^ Disinformation complicates the task by contributing contradiction and distortion. Medical jargon obscures understanding.^226^ Population preferences and access to technology raise questions about targeting and equity. The internet and social media have greatly influenced how the public consumes information. Although the medical community has utilized social media to mobilize the public in healthcare topics,^227^ more information is needed into the effectiveness of these approaches.

### Occupational safety

The two research domains identified below (also see Supplementary Materials, Suggested Methodologies, Table 4) represent priority areas with unanswered research questions in occupational safety:

- *Priority area 1:* Identify strategies that optimize occupational health protections, including during public health crises and incident management.
- *Priority area 2:* Determine effective approaches to prevent presenteeism while also preventing staffing shortages.

The General Duty Clause of the Occupational Safety and Health Act of 1970^228^ requires employers to provide workers with a safe workplace that does not have known hazards that cause or are likely to cause death or serious injury.^229,230^ In acute care settings, these activities are often assigned to the occupational health department, and administered in conjunction with the hospital epidemiologist, the Infection Prevention and Control Program, and the human resources department.

Research is needed to find ways to improve coordination among these groups during emergency preparedness and response.^231^ In settings with insufficient staffing and physical resources and few or no dedicated occupational health staff (e.g., pediatrics, long-term care, home care, ambulatory care, etc.), research also can help to identify opportunities to adopt or adapt existing resources through virtual platforms, collaboratives, training for frontline HCP, consultation support, or other approaches.

Another occupational health research priority is the safety problem of presenteeism, defined as the act of attending work while ill and potentially infectious to others,^232^ involving patient safety, worker safety, and staffing adequacy. In the 2019 revision of the guideline *Infection Control in Healthcare Personnel*, CDC recommends that organizations’ sick leave policies encourage HCP to report potentially infectious exposures or illnesses, appropriately use sick leave, and adhere to work restrictions,^232^ and for organizations to provide timely, confidential, and non-punitive mechanisms for HCP to report potential exposures and services. Lack of access to paid sick leave is more common among low-wage employees in non-acute and under-resourced settings such as nursing homes and home health care.

To understand beliefs and organizational factors that contribute to presenteeism and how to change them, researchers might pursue qualitative and/or quantitative studies: epidemiologic studies of the prevalence of presenteeism, large multicenter qualitative studies to explore the rationale behind presenteeism, and national surveys to understand current sick leave policies. Such studies would contribute data that could inform local and national best practices and evidence-based guidelines for standard measurement of presenteeism, optimal staffing models, approaches to symptom screening, and other factors.

## Evolving research topics

In the context of dynamic local and global conditions, we have identified evolving topics for researchers and funders to consider for HAI prevention. We view this agenda as a call to action after the COVID-19 pandemic. First, the US population is becoming increasingly diverse. Racial and ethnic minorities and other groups typically are under-represented in research due to barriers that include study design (e.g., studies routinely exclude non-English speaking patients), financial (such as lack of insurance limiting their access to healthcare enterprise, lack of transportation), geographic (rural vs. urban), communication and cultural barriers (such as lack of trust based on prior experiences). Gender identity as a social determinant of health also is underexplored. Principles of diversity, equity, and inclusion should inform research programs, incorporating diverse factors as key variables, and allocating appropriate time and resources to address barriers to research participation.

Second, the COVID-19 pandemic has shown us that various entities within a community or a region, such as community health organizations, schools, colleges, and businesses no longer can operate in silos. Engagement with these entities is essential to enhancing the public health infrastructure, with investigation into models and tools, high-priority topics, and ways to engage students in health sciences and public health at earlier stages of their careers. Such engagement and workforce development can help enhance overall health of the community, improve public health response to various outbreaks, improve uptake of interventions and reduce misinformation.

Third, COVID-19 has shown us that countries can no longer operate in silos. We live in an interdependent global era. Policies, politics, and priorities in one country directly impact populations in other countries. *There can be no global economy without global public health*. Researchers must creatively propose—and funders should support—research initiatives that allow us to study populations and infection prevention infrastructures across different countries and populations. Fourth, use of artificial intelligence in health care is inevitable. Developing frameworks and models of their use within infection prevention and healthcare epidemiology is prudent, timely and an evolving area of research. Lastly, we must consider the impact of global climate change on the epidemiology of HAIs. We view this agenda to be a call to action as we prepare for a world after the COVID-19 pandemic.

## Supporting information

Kwon et al. supplementary material 1 Kwon et al. supplementary material

Kwon et al. supplementary material 2 Kwon et al. supplementary material

## Supplementary material

The supplementary material for this article can be found at https://doi.org/10.1017/ice.2024.125.
