# Supplementary material for: A call to action: the SHEA research agenda to combat healthcare-associated infections
Source: Infect Control Hosp Epidemiol. 2024 Sep;45(9):1023–40. doi: 10.1017/ice.2024.125 (PMC11518679; doi:10.1017/ice.2024.125)
Supplement: Kwon et al. supplementary material 1 — Kwon et al. supplementary material [file S0899823X24001259sup001.docx]

The SHEA Research Agenda to Combat HAIs: Supplementary Materials

Contents

[**SHEA Research Network (SRN) Research Priorities Survey Results** 1](#_Toc156995507)

[Legend 1](#_Toc156995508)

[Table 1: Priorities by Topic Area 1](#_Toc156995509)

[Table 2: Priorities by Research Question 2](#_Toc156995510)

[Table 3: Priorities by Topic Area: Populations and Settings 10](#_Toc156995511)

[Table 4: Priorities by Research Question: Populations and Settings 10](#_Toc156995512)

# **SHEA Research Network (SRN) Research Priorities Survey Results**

## Legend

| Green | Top 5 priorities per survey question grouping |
| --- | --- |

## Table 1: Priorities by Topic Area

| **Count** | | **52** |
| --- | --- | --- |
| **Total Response Rate**   (2021 survey requests sent: Nov. 26, Nov. 11, Oct. 6) | | **57%** |
| **18. Topics of greatest need for sustained, focused, and funded research? Max 5.** | **selections/**  **respondents to question** | **%** |
| Implementation science in healthcare epidemiology and infection prevention | 34/49 | 69.4% |
| Modes of transmission of pathogens in healthcare settings | 27/49 | 55.1% |
| Diagnostics and testing | 25/49 | 51.0% |
| Infection prevention during public health emergencies | 20/49 | 40.8% |
| How to use data to prevent HAIs | 19/49 | 38.8% |
| Role of the environment in colonization of patients and healthcare personnel (HCP) | 17/49 | 34.7% |
| Environmental hygiene | 17/49 | 34.7% |
| HCP burnout/wellness | 14/49 | 28.6% |
| Educating and informing the public | 11/49 | 22.4% |
| Device-associated infections | 10/49 | 20.4% |
| Role of the community in the spread of infections in healthcare | 8/49 | 16.3% |
| Role of social interaction/engagement for patients | 5/49 | 10.2% |
| Reporting to public health | 4/49 | 8.2% |
| Occupational safety | 3/49 | 6.1% |
| **Other - Write In:**   - Accurate diagnosis - Sustainability - Infection prevention during public health emergencies - Educating HCP in IP | 4/49 | 8.2% |
| **Written comments:**   - Too much non-evidence-based shift towards single-use disposables.  We need to figure out how to mandate design for safe cleaning and reuse, to build supply chain resilience in a resource constrained world, and to reduce healthcare pollution in the name of infection prevention to the complete exclusion of harm to the communities we serve. - Lots of technologies for sale.  Knowing which ones, their risks/benefits/tradeoffs is huge.  Stay with BioFire or implement T2?  What is the ROI? - How to use data, meaning how to match HAI definitions to reflect value and quality of care (not use surveillance definitions for quality assessment) - Many areas, including HCP burnout, are critically important, but we don't need to study them further to know they are a genuine threat. - Sequencing is becoming a very important part of hospital epi/IP especially in determining the role of the healthcare environment towards transmission of pathogens to patients. Sequencing costs money and this can be a rate limiting factor. - Given the epidemic of HCP burnout, it is essential to study the impacts of this on adherence to IPC measures - There is a gap in knowledge about transmission of infection in hospital and the role of environment on colonization of patients by MDRO. - Pick a winner: all have a degree of importance at various times. Picked items that have more to do with community involvement and education, as this is the 'next step' in Infection Prevention (IP). The number of misconceptions as to what we do and how it affects our communities is large. With COVID and publicly reportable illness / diseases, IP role in public health is now becoming more visible and if done properly, can be beneficial to more than just our hospital settings - Role of the community in the spread of infections in healthcare | | |

## Table 2: Priorities by Research Question

| **20-34. To what extent would care improve if these questions had well-researched answers?**  1=lowest, 9=highest | **Research question** | **Response #/Response # from 18** | **% response to question** | **% response to 18** (N=49) | **Mean** (1-9) | **Median** (1-9) | **By topic area, highest to lowest priority** **research question** (response*mean) |
| --- | --- | --- | --- | --- | --- | --- | --- |
| **20. modes of transmission in healthcare settings** | What interventions interrupt specific types of transmission pathways? | 26/29 | 89.7% | 53.1% | 8.1 | 8.5 | 211.0 |
|  | What methods prevent in-hospital acquisition of multidrug-resistant pathogens? | 28/29 | 96.6% | 57.1% | 7.3 | 7 | 203.3 |
|  | What are effective infection prevention practices for bedside procedures? | 28/29 | 96.6% | 57.1% | 6.1 | 6 | 172.1 |
| **21. role of the environment** | To what extent, and how long can a hospital's microbial environment colonize patients? | 18/18 | 100.0% | 36.7% | 6.9 | 7 | 123.9 |
|  | How can hospitals evaluate multidrug-resistant organisms (MDROs) or C. difficile in readmitted or long-hospital-stay patients to understand colonization's contribution to infection? | 18/18 | 100.0% | 36.7% | 6.7 | 7 | 121.0 |
|  | Do patients change the hospital's microbial environment? | 17/18 | 94.4% | 34.7% | 5.5 | 6 | 94.0 |
| **22. environmental hygiene** | What is the role of environmental contamination in patients' acquisition of HAIs? | 15/16 | 93.8% | 30.6% | 7.4 | 8 | 111.0 |
|  | How can hospitals conduct long-term observation of the environment? | 16/16 | 100.0% | 32.7% | 6.9 | 7 | 110.0 |
|  | What is the effectiveness of long-term microbial cultures and microbial testing of the environment? | 16/16 | 100.0% | 32.7% | 5.9 | 6.5 | 95.0 |
|  | What percentage of MDRO infection and CDI is caused by environmental contamination? | 16/16 | 100.0% | 32.7% | 5.9 | 6 | 95.0 |
|  | What is the role of serial testing vs. clinical surveillance of patients? | 16/16 | 100.0% | 32.7% | 5.8 | 5.5 | 93.0 |
| **23. HCP burnout** | How does constrained staffing or other resources affect the implementation and sustainability of existing and new HAI prevention strategies? | 14/14 | 100.0% | 28.6% | 8.1 | 8 | 113.0 |
|  | What is the role of risk assessments and readiness scales to ascertain workforce capacity for HAI prevention (i.e., burnout, staffing changes, what units can reasonably undertake)? | 14/14 | 100.0% | 28.6% | 6.6 | 7 | 93.0 |
|  | What strategies optimize occupational health protections, including during incident management? | 14/14 | 100.0% | 28.6% | 6.1 | 7 | 86.0 |
| **24. occupational safety** | What is the role of risk assessments and readiness scales to ascertain workforce capacity for HAI prevention (i.e., burnout, staffing changes, what units can reasonably undertake)? | 3/3 | 100.0% | 6.1% | 6.3 | 6 | 19.0 |
|  | What are ways to prevent presenteeism while also preventing staffing shortages? | 3/3 | 100.0% | 6.1% | 6.3 | 7 | 19.0 |
|  | What strategies are needed to optimize occupational health protections, including during crises, e.g. public health emergencies? | 3/3 | 100.0% | 6.1% | 6.0 | 6 | 18.0 |
|  | What HCP education is needed to improve prevention, recognition, and reporting of MDROs? | 3/3 | 100.0% | 6.1% | 5.3 | 7 | 16.0 |
|  | How does constrained staffing or other resources affect the implementation and sustainability of existing and new HAI prevention strategies? | 3/3 | 100.0% | 6.1% | 8.0 | 9 | 24.0 |
| **25. infection prevention during public health emergencies** | What is the impact of public health emergencies on the epidemiology of HAIs? | 20/20 | 100.0% | 40.8% | 7.8 | 8 | 156.0 |
|  | How does constrained staffing or other resources affect the implementation and sustainability of existing and new HAI prevention strategies? | 20/20 | 100.0% | 40.8% | 7.6 | 8 | 152.0 |
|  | What strategies optimize occupational health protections, including during crises, e.g. public health emergencies? | 20/20 | 100.0% | 40.8% | 6.9 | 7 | 137.0 |
|  | How can hospitals prevention MDROs during public health emergencies? | 20/20 | 100.0% | 40.8% | 6.8 | 7 | 135.0 |
|  | What is the role of risk assessments and readiness scales to ascertain workforce capacity for HAI prevention (i.e., burnout, staffing changes, what units can reasonably undertake)? | 20/20 | 100.0% | 40.8% | 6.4 | 7 | 128.0 |
| **26. how to use data to prevent HAIs** | What are the minimum and the optimum levels of resources, both staffing and technical, needed to achieve ideal quality outcomes? | 18/18 | 100.0% | 36.7% | 8.1 | 8.5 | 146.0 |
|  | What are the operational, financial, and reputational costs of HAIs that justify optimal levels of resources? | 18/18 | 100.0% | 36.7% | 7.3 | 8 | 131.0 |
|  | How can hospitals identify where noise, gaming the system, and interpretation obscure quality metrics? | 18/18 | 100.0% | 36.7% | 7.1 | 7 | 128.0 |
|  | What effective epidemiology or statistical approaches can more definitively answer healthcare epidemiology questions? | 18/18 | 100.0% | 36.7% | 6.8 | 7 | 122.0 |
|  | How can HAI-prevention be presented as a business case and prioritize with other hospital safety initiatives? | 18/18 | 100.0% | 36.7% | 6.7 | 7.5 | 121.0 |
|  | What is the potential to develop meaningful harm measures across related practices (IP, ASP, diagnostic stewardship) to inform frontline work and obtain administrative support? | 18/18 | 100.0% | 36.7% | 6.6 | 7 | 119.0 |
|  | How can hospitals collect long term data on MDRO colonization and development of HAIs? | 18/18 | 100.0% | 36.7% | 6.5 | 6.5 | 117.0 |
|  | How can hospitals maximize the signal to noise ratio in communicating about HAI when the number of events is small/infrequent? | 18/18 | 100.0% | 36.7% | 6.0 | 6 | 108.0 |
| **27. the role of the community** | What is the prevalence of antimicrobial resistance outside of the hospital, e.g. in the community, nursing homes, communal living settings? | 8/8 | 44.4% | 16.3% | 7.3 | 7.5 | 58.0 |
|  | What is the epidemiology of HAIs in the community? | 8/8 | 100.0% | 16.3% | 6.8 | 7.5 | 54.0 |
| **28. diagnostics and testing** | How does diagnostic stewardship affect HAIs? | 25/25 | 100.0% | 51.0% | 7.7 | 8 | 193.0 |
|  | What measures should be used? | 25/25 | 100.0% | 51.0% | 7.2 | 8 | 179.0 |
|  | What is the role of interdisciplinary involvement and education in diagnostic stewardship? | 24/25 | 96.0% | 49.0% | 6.5 | 7 | 155.0 |
| **29. device-associated infections** | What strategies are effective to reduce CLABSI, including MBI-CLABSI in oncology patients, where central line use is common? | 9/9 | 100.0% | 18.4% | 7.3 | 8 | 66.0 |
|  | Using the current NHSN definitions, what is the morbidity and mortality of ventilator-associated events, ventilator-associated infectious conditions, and possible ventilator-associated pneumonia? | 9/9 | 100.0% | 18.4% | 5.0 | 6 | 45.0 |
| **30. the role of social interaction/engagement for patients** | What are the costs and benefits of common isolation measures? | 4/4 | 100.0% | 8.2% | 6.8 | 7 | 27.0 |
|  | How do visitation policies and related infection prevention measures affect in-hospital transmissions in pediatric and neonatal settings? | 4/4 | 100.0% | 8.2% | 6.3 | 6.5 | 25.0 |
|  | How do visitation policies affect adult and pediatric HAI rates? | 4/4 | 100.0% | 8.2% | 6.0 | 6.5 | 24.0 |
| **31. implementation** | What strategies/techniques facilitate behavior change in the healthcare workspaces? | 34/35 | 97.1% | 69.4% | 7.8 | 8.5 | 266.0 |
|  | How can hospitals build sustainability into infection prevention interventions? How can they measure their effects? | 34/35 | 97.1% | 69.4% | 7.6 | 8 | 257.0 |
|  | What are the minimum and the optimum levels or resources, both staffing and technical, needed to achieve ideal quality outcomes? What are the operational, financial, and reputational costs of HAIs that justify these resources? | 35/35 | 100.0% | 71.4% | 7.3 | 8 | 254.0 |
|  | How well do evidence-based recommendations, e.g. national guidelines, translate into real-world practices? Is the rate of translation and uptake measurable? Does it vary based on setting? Are they sustained? | 34/35 | 97.1% | 69.4% | 7.1 | 7.5 | 243.0 |
|  | What, beyond current bundles, can prevent harm from HAIs? | 34/35 | 97.1% | 69.4% | 7.0 | 8 | 237.0 |
|  | How can hospitals identify where noise, gaming the system, and interpretation obscure the quality metric? | 33/35 | 94.3% | 67.3% | 6.5 | 7 | 215.0 |
|  | What HCP education is needed to improve prevention, recognition, and reporting of MDROs? | 35/35 | 100.0% | 71.4% | 5.8 | 6 | 202.0 |
| **32. public health reporting** | What HCP education is needed to improve prevention, recognition, and reporting of MDROs? | 4/4 | 100.0% | 8.2% | 5.8 | 6 | 23.0 |
|  | What is the burden of reportable HAIs and MDROs in rural settings? Are they accurate? | 4/4 | 100.0% | 8.2% | 5.5 | 6 | 22.0 |
|  | To what extent do hospitals learn of MDRO transmissions? | 4/4 | 100.0% | 8.2% | 6.0 | 5.5 | 24.0 |
|  | How can hospitals maximize the signal to noise ratio, when the number of HAI events is small/infrequent? | 4/4 | 100.0% | 8.2% | 7.0 | 7.5 | 28.0 |
| **33. educating and informing the public** | What are the best ways for public health to recognize and combat disinformation? | 11/11 | 100.0% | 22.4% | 8.4 | 8 | 92.0 |
|  | How can HCP and healthcare organizations effectively communicate and advocate for evidence-based health recommendations? | 11/11 | 100.0% | 22.4% | 8.0 | 9 | 88.0 |
| **Other - Write In:** |  | | | | | | |
| **20. modes of transmission in healthcare settings** | Update the indications for transmission-based precautions. | | | | | | |
|  | Resource conservation through more effective measures, and protect public health from pollution and supply chain risks | | | | | | |
|  | What novel surface and environmental modifications can interrupt transmission | | | | | | |
|  | Clarity from CDC on modes of transmission and standard precautions | | | | | | |
| **21. role of the environment** | How many of the previous epidemiological links to the environment can be confirmed with modern testing (for example, whole genome sequence)? | | | | | | |
|  | Improve methods of disinfection of the environment | | | | | | |
| **22. environmental hygiene** | Again, would conserve resources through more efficient and effective measures, and protect public health from pollution and supply chain risks. | | | | | | |
|  | Mold surveillance | | | | | | |
|  | Water safety | | | | | | |
|  | What is a reliable, standardized, informative way to measure environmental contamination and correlation to patient risk? | | | | | | |
| **23. HCP burnout** | Financial impact of HAIs due to burnout in frontline staff and IPs | | | | | | |
| **25. infection prevention during public health emergencies** | Best training strategies for frontline staff across healthcare settings | | | | | | |
|  | How can readiness (both staff and supply) readiness be sustained? | | | | | | |
|  | Improve staff training | | | | | | |
| **26. how to use data to prevent HAIs** | Address shared decision-making around appropriate resource stewardship, not about withholding resource or increasing risk to avoid tests/treatments and confer reputational and medicolegal buffer. | | | | | | |
|  | Epidemiologists should lead, rather than follow, the drive to better understanding of HAI measures. | | | | | | |
|  | How well do surveillance definitions reflect actual care quality? | | | | | | |
|  | Validity and reliability of publicly reported measures used for payment | | | | | | |
| **27. the role of the community** | What are community sources of significant pathogens, particularly C. difficile? | | | | | | |
|  | What transmission pathways exist for MDROs between community/long-term care, emergency response vehicles, and the hospital setting | | | | | | |
| **28. diagnostics and testing** | Surveillance testing for emerging fungal pathogens, sequencing | | | | | | |
|  | Differentiate between colonization and infection when using molecular diagnostics and syndromes panels | | | | | | |
|  | How can "diagnostic preparedness" be assured for future public health emergencies | | | | | | |
| **29. device-associated infections** | How can devices and their surfaces be redesigned to prevent infections | | | | | | |
|  | What additional evidence-based bundle elements could impact CAUTI rates? | | | | | | |
| **30. the role of social interaction/engagement for patients** | What is the true impact of Speak UP campaigns to improve hand hygiene or other infection practices | | | | | | |
| **31. implementation** | Identify emerging infections | | | | | | |
|  | What strategies facilitate behavior change gets 18 stars! | | | | | | |
| **33. educating and informing the public** | How can we assure the medical / ID profession is the communicator rather than the politician (of physician-turned-politician | | | | | | |
|  | another way of saying "how can we de-politicize public health communication" needs 15 stars | | | | | | |
| **Written comments:**   - The most pressing question is how many FTE for Infection Diseases Healthcare Epidemiologists are needed/hospital or healthcare system?  How many for Stewardship should be minimum? - We need high-quality, well-designed studies to support many of our long-time default practice. We need to have guidelines that venture into areas where the data is sparse.  Much of healthcare is shifting to the outpatient space, where almost no Infection Prevention & Control structure exists. (like IV therapy, surgeries without admissions). How can this harm be avoided? - Implementation science and change management are really important. It's so hard to identify one best practice and sustain its implementation over time. | | | | | | | |

## Table 3: Priorities by Topic Area: Populations and Settings

| **35. Populations or settings is there the greatest need for sustained, focused, and funded research?** **Max 3.** | **selections/**  **respondents to question** | **% respondents** |
| --- | --- | --- |
| Acute care hospitals** | 30/48 | 62.5% |
| Nursing homes | 29/48 | 60.4% |
| Ambulatory/outpatient | 17/48 | 35.4% |
| Minority, under-represented, and/or socio-economically disadvantaged populations | 16/48 | 33.3% |
| Resource-limited settings | 15/48 | 31.3% |
| Immunocompromised patients | 7/48 | 14.6% |
| Pediatrics | 7/48 | 14.6% |
| Home healthcare | 6/48 | 12.5% |
| Other - Write In | 5/48 | 10.4% |

*** “Acute care hospitals” was added as an answer selection during testing and does not have any specific research questions below.*

## Table 4: Priorities by Research Question: Populations and Settings

| **36-43. To what extent would care improve if these questions had well-researched answers?** 1=lowest, 9=highest | **Research question** | **Response #/Response from 35** | **% response to question** | **% response to 35** (N=49) | **Mean** (1-9) | **Median** (1-9) | **By topic area, highest to lowest priority research question** (response*mean) |
| --- | --- | --- | --- | --- | --- | --- | --- |
| **36. ambulatory or outpatient settings** | What methods are reliable in detecting post-discharge surgical site infections in ambulatory patients? | 17/30 | 56.7% | 34.7% | 6.1 | 6 | 104.3 |
|  | What are best practices to monitor and improve HH and other IP interventions in ambulatory settings? | 2/30 | 6.7% | 4.1% | 8.0 | 8 | 16.0 |
|  | What role does the ambulatory setting have in transmission of MDRO? | 2/30 | 6.7% | 4.1% | 8.0 | 8 | 16.0 |
|  | How can risk of HAI with ambulatory device use (lines) or care processes be reduced? | 2/30 | 6.7% | 4.1% | 6.0 | 6 | 12.0 |
| **37. resource-limited settings** | What is the effect of limited resources (e.g. staffing) on the implementation and sustainability of HAI prevention strategies? | 15/15 | 100.0% | 30.6% | 8.0 | 8 | 120.0 |
|  | What are effective methods to implement and sustain evidence-based recommendations in resource-limited settings? How can uptake and outcomes of these recommendations be measured, especially among small sample sizes (e.g. rural)? | 15/15 | 100.0% | 30.6% | 7.9 | 9 | 119.0 |
|  | What is the burden of HAIs and MDROs in rural areas? Are they reported? Are public health agencies informing hospitals of these reports? | 15/15 | 100.0% | 30.6% | 6.0 | 7 | 90.0 |
| **38. pediatrics** | What is the prevalence of non-device associated HAIs in children (including respiratory viral infections)? | 7/7 | 100.0% | 14.3% | 7.9 | 8 | 55.0 |
|  | What metrics/methods should be used to track hospital-onset bacteremia in children? | 7/7 | 100.0% | 14.3% | 7.1 | 7 | 50.0 |
|  | What are effective and sustainable practices to decrease pediatric HAIs? | 7/7 | 100.0% | 14.3% | 7.1 | 7 | 50.0 |
|  | What metrics/methods should be used to track pediatric SSIs? | 7/7 | 100.0% | 14.3% | 6.3 | 7 | 44.0 |
| **39. nursing homes** | What successful interventions have been used in nursing homes that are practical, easily reproduced by any size facility and not costly to prevent MDRO transmission? | 29/29 | 100.0% | 59.2% | 7.8 | 8 | 226.0 |
|  | How can non-skilled nursing facility long term care facilities (e.g., assisted living, adult family homes) have access to IP and HAI prevention resources (including awareness and education)? | 29/29 | 100.0% | 59.2% | 7.3 | 8 | 213.0 |
| **40. home healthcare** | How can HAIs be monitored in home healthcare? | 6/6 | 100.0% | 12.2% | 7.8 | 8 | 47.0 |
|  | What are methods for home healthcare HAI prevention for the individual patient and their informal caregivers? For home health staff? | 6/6 | 100.0% | 12.2% | 7.0 | 7 | 42.0 |
| **41. immunocompromised patients** | What strategies are effective to reduce CLABSI, including MBI-CLABSI in oncology patients, where central line use is common? | 6/7 | 100.0% | 14.3% | 7.7 | 9 | 46.3 |
|  | What strategies are effective to reduce the risk of CDI in immunocompromised hosts, particularly in transplant patients and those receiving chemotherapy? | 6/7 | 85.7% | 12.2% | 7.7 | 8 | 46.0 |
| **42. minority, under-represented, and/or socio-economically disadvantaged populations** | What are the reported disparities in HAI and MDRO rates in socio-economically disadvantaged populations? | 15/15 | 100.0% | 30.6% | 7.7 | 8 | 115.0 |
| **Other - Write In:** |  | | | | | | |
| **36. ambulatory or outpatient settings** | Transmission of MDROs in outpatient settings (particularly longer-stay ambulatory settings such as chemotherapy infusion) | | | | | | |
|  | How can pathogen transmission between acute care facilities be detected and interrupted?  How can improved and standardized inter-facility communication be implemented? | | | | | | |
| **37. resource-limited settings** | The world is resource constrained, whether we feel it or not. E.g., taking a third vaccine in the US while less than 10% of the global south is vaccinated against Covid ensures the virus will live and mutate and there will be continued demand for globally | | | | | | |
|  | What are the high priority interventions? How we measure the impact? | | | | | | |
| **41. immunocompromised patients** | Duration of isolation for MDROs important as well. | | | | | | |
|  | How can we prevent mucosal barrier injury BSI? | | | | | | |
| **42. minority, under-represented, and/or socio-economically disadvantaged populations** | Effective strategies to reduce disparities. | | | | | | |
|  | How do providers modulate infection prevention practices to improve patient outcomes from disadvantaged populations? | | | | | | |
| **Written comments:**   - resource limited and socioeconomically disadvantaged are similar issues.  Leadership needs to be funded to arise from within these communities; outside leadership and entitlement programs are counterproductive in the long run - All of the HAIs mentioned here refer to the ones defined by NHSN. Unfortunately, NHSN is not perfect as we all understand. However, sometimes, the flaws in the NHSN definitions are so obvious that make everyone to feel the infection is simply caught by the NHSN definition. These scenarios simply hinder our ability to engage the clinical and frontline staff for interventions. We tried to reach NHSN via the only way provided by NHSN, the email, for clarification. We received responses from the NHSN staff that doesn't make any sense and makes us to wonder if NHSN staff is well trained on performing the job. I wish NHSN can work with us and continue establishing itself as the respected sole authority in classifying HAIs. - We are probably missing some big opportunities by only having one question here (SHEA staff note: respondent row 29; unclear to which selection they are referring) - I provide consultative services to a LTC Facility.  Staff turnover and training of the IP is a huge weak link. - The future will be better and safer than today if we can work on these problems. - How can we prevent healthcare-associated BSI in patients without central catheters? - Any focus on long term care (SNFs but also AL and AFHs, as well as rural health care providers (including CAHs) would be important. The pandemic has shed light on the disparities in these settings. | | | | | | | |
