# Supplementary material for: A call to action: the SHEA research agenda to combat healthcare-associated infections
Source: Infect Control Hosp Epidemiol. 2024 Sep;45(9):1023–40. doi: 10.1017/ice.2024.125 (PMC11518679; doi:10.1017/ice.2024.125)
Supplement: Kwon et al. supplementary material 2 — Kwon et al. supplementary material [file S0899823X24001259sup002.docx]

The SHEA Research Agenda to Combat HAIs: Supplementary Materials

Contents

[**Suggested Methodologies, Populations, and Intended Outcomes for Research Priorities** 1](#_Toc156995109)

[Table 1: Implementation science in healthcare epidemiology (HE) and infection prevention (IP), modes of transmission, and diagnostic stewardship 1](#_Toc156995110)

[Table 2: IP during public health emergencies (PHE), how to use data to prevent HAIs, and the role of the environment 4](#_Toc156995111)

[Table 3: HCP wellness and burnout, device-associated infections, and the role of the community in the spread of infections in healthcare 9](#_Toc156995112)

[Table 4: The Role of Social Interaction and Engagement for the Public, Patients, and Visitors, Occupational Safety 18](#_Toc156995113)

[Table 5: Post-Acute Care and Nursing Homes, Pediatrics 24](#_Toc156995114)

# **Suggested Methodologies, Populations, and Intended Outcomes for Research Priorities**

## Table 1: Implementation science in healthcare epidemiology (HE) and infection prevention (IP), modes of transmission, and diagnostic stewardship

| **Priority Area** | **Research Question** | **Clinical Rationale** | **Suggested Methodology/**  **Population** | **Intended Outcomes** |
| --- | --- | --- | --- | --- |
| **1. Implementation Science in HE and IP** | | | | |
| 1. Behavior change strategies | What strategies/techniques facilitate behavior change in healthcare workspaces? | Theory-based behavior change interventions and techniques can help improve infection prevention and control practices. | Mixed methods, quasi-experimental, stepped wedge designs | Improved uptake and use of evidence-based infection prevention practices. |
| 2. Strategies to support sustainability | How can hospitals and other healthcare settings build sustainability into infection prevention interventions? | Effectively implementing a practice or intervention in a given practice setting also requires establishing strategies to ensure that the gains of practice implementation are sustained. | Mixed methods, quasi-experimental, stepped wedge designs | Ongoing use of recommended infection prevention practices to ensure safe, high quality care delivery. |
| 3. Extent of implementation across settings | 3a. How well do evidence-based recommendations, e.g., national guidelines, translate into real-world practice? | Implementation science, both implementation research and implementation practice, are critical for addressing inherent challenges to the delivery of evidence-based infection prevention practices across healthcare settings and ensuring safe, effective care. | Observational | Develop a better understanding of areas for enhanced focus and strategies to facilitate implementation across different healthcare settings. |
|  | 3b. Is the rate of translation and uptake measurable? Does it vary based on setting? Are practice changes sustained? |  |  |  |
|  | 3c. What is the effect of limited resources (e.g., staffing) on the implementation and sustainability of healthcare-associated infection (HAI) prevention strategies? |  |  |  |
| **2. Modes of Transmission** | | | | |
| 1. Defining infection chains using sequencing data | 1a. How common is transmission in the hospital? | While known HAIs are common, they likely only represent a fraction of actual transmissions as colonized individuals are rarely identified | Regular surveillance cultures and whole genome sequencing of positive cases. | Information that leads to development of strategies to control pathogens in healthcare facilities. |
|  | 1b. What is the source of HAIs in the hospital? | Attributable sources of colonization and infection in the hospital are rare. | Whole genome sequencing of pathogens collected through routine clinical samples | Determine if whole genome sequencing of all infections can provide timely or useful information to inform healthcare infection prevention strategies. |
| 2. Role of healthcare personnel (HCP) in transmission | 2a. To quantify the relative role of direct versus indirect transmission | Understanding the relative contribution that HCP play in directly moving pathogens between two patients compared to indirect transmission through environmental contamination | Prospective cohort studies to determine the transfer of pathogens from HCP to patients and environmental surfaces. Randomized trials of cleaning interventions that include patient infection outcomes | Determine how often patients are infected by pathogens from the HCP transmitting from other patients or from the healthcare environment |
|  | 2b. How well does EHR data capture patient connected networks | Patients have limited contact with other patients, but HCWs have significant contact with multiple patients. Nearly all healthcare facilities have EHRs. How well does the EHR data capture connectivity and transmissions between patients? | Prospective cohort studies to match detailed observation data of HCW movement with EHR data and patient infection outcomes. | Identify hidden networks of transmission in the hospital |
| **3. Diagnostic Stewardship** | | | | |
| 1. Sociobehavioral, contextual, and adaptive factors | What are the sociobehavioral, contextual, and adaptive factors that impact test use? | Improve test use by understanding sociobehavioral, cultural, and adaptive barriers and facilitators; Increase uptake of implementation science frameworks like COM-B model, CFIR, etc. | Mixed methods studies, qualitative studies, implementation studies to identify specific sociobehavioral, cultural, and adaptive barriers and facilitators that impact testing | Outcomes of qualitative or observational work may produce an understanding of which barriers impact test use, and help design focused interventions to target specific barriers |
| 2. Continuum of testing | What opportunities exist for diagnostic stewardship across the continuum of testing? | Traditionally, most diagnostic stewardship interventions have focused on the ordering (pre-analytic) phase of testing, missing out on other phases of testing- collection, processing and reporting, as well as include non-culture-based tests | Retrospective, quasi-experimental, and implementation studies of diagnostic stewardship strategies focused on different phases of testing- collection, ordering, processing and reporting, as well as include non-culture-based tests | Develop interventions for diagnostic stewardship across the continuum of testing, and focus on low hanging fruit (e.g., urine contamination) |
| 3. Non-culture—based tests | What opportunities exist for diagnostic stewardship for non-culture-based tests? | Beyond interventions focused on cultures, there is a need to address non-culture precursor tests like urinalysis, and newer tests like procalcitonin and molecular tests | Retrospective, quasi-experimental, and implementation studies of diagnostic stewardship strategies focused on different phases of testing- collection, ordering, processing, and reporting, as well as include non-culture-based tests | Better understanding of cost effectiveness, appropriate utility and setting, long-term outcomes, and sustainability of non-culture-based testing |
| 4. Clinical decision support | How can we leverage the electronic health record (EHR) to support diagnostic stewardship interventions | Understand how to leverage the EHR for diagnostic stewardship intervention | Quasi-experimental, and implementation studies to evaluate EHR based interventions for diagnostic stewardship | Assess sustainability of EHR based interventions and assess these interventions for unintended consequences. |
| 5. Future metrics | How can we develop more meaningful performance metrics? | Develop performance metrics that align with diagnostic stewardship interventions and provide meaningful information back to facilities to improve outcomes | Feasibility and pilot studies of novel or proposed metrics | Creation of novel metrics that reflect infectious and noninfectious complications and align with quality improvement efforts that improve patient outcomes. For example: using measures that focus on diagnostic test utilization (i.e., urine cultures collected/1000 patient days in addition to CAUTI rates) |

## Table 2: IP during public health emergencies (PHE), how to use data to prevent HAIs, and the role of the environment

| **Priority Area** | **Research Question** | **Clinical Rationale** | **Suggested Methodology/**  **Population** | **Intended Outcomes** |
| --- | --- | --- | --- | --- |
| **4. IP During Public Health Emergencies** | | | | |
| 1. How can hospitals control multidrug-resistant organisms (MDROs) during public health emergencies? | 1a. How should personal protective equipment (PPE) be prioritized during public health emergencies (PHE) when the demand is high? Is there a hierarchy for which MDROs needs to be prioritized for PPE use? | Shortages of PPE during COVID-19 pandemic caused rationing and extended use. Additionally, the effects on respirators of various disinfection methods have yet to be fully researched and proven safe and effective. The effectiveness of KN95 respirators is largely unknown at this time. Some acute care facilities decided not to place patients colonized with some MDROs in isolation precautions due to PPE shortages. This was done at a facility level based on limited local epidemiology data, rather than an overall research-based approach. | Before/after (intervention) studies  Literature review | Decrease shortages of PPE during PHE. |
|  | 1b. Is antimicrobial stewardship during public health emergencies different from antimicrobial stewardship in stable times? | Different prescribing patterns may occur during a PHE based on the organism involved. | Comparison of pandemic and pre-pandemic antimicrobial usage | Determine if the focus of AMS programs must change during PHEs.  Identify antimicrobial stewards in healthcare settings beyond infectious disease physicians and pharmacists. |
| 2. How does constrained staffing or other resources affect the implementation and sustainability of existing and new HAI prevention strategies? | 2a. Can infection prevention practices such as PPE donning and doffing, and placement of devices such as a central line, occur correctly without a trained observer or a checklist? Can technology be used to 'monitor' these procedures? | Studies have indicated a high percentage of HCP contaminate themselves when doffing PPE. Staffing shortages may preclude having a trained observer available during PHE. | Feasibility/ proof-of-concept studies followed by well-designed randomized controlled clinical trials | Decrease the likelihood of HCP improperly wearing PPE or contamination during doffing. Decrease the likelihood of device-associated infection. |
|  | 2b. Can PPE be used beyond their expiration date when resources are limited? What is the impact of reprocessing single-use PPE? | The science behind setting expiration dates is not clear. PPE shortages during PHE may compel use of items beyond expiration dates and the safety of these practices are unknown. | Laboratory-based experiments | Improved stewardship of PPE supplies during PHE without compromising safety. |
|  | 2c. How do we safely prioritize/triage infection prevention activities when a change in focus changes due to a public health emergency? What is the optimal infection prevention staffing during public health emergencies? Can non-clinical staff be utilized to augment infection prevention staffing during PHE? | During PHE, infection prevention and healthcare epidemiology personnel-time is a precious resource. | Semi-structured interviews, Sensemaking approaches, Systematic reviews. Innovation is critically needed in this area. | Better stewardship of personnel-time in infection prevention and healthcare epidemiology. Identification of alternative personnel who can step in for delegation of tasks previously within their span of responsibility. |
|  | 2d. How do we effectively compile lessons learned during natural disasters or infectious disease public health emergencies? What is the necessary infrastructure for enabling effective and timely data collection when an emergency occurs? | Infection preventionists and healthcare epidemiologists appreciate ready-to-use guidance during PHE and seeing that lessons learned during previous disasters are effectively integrated into approach to ongoing disaster(s). | Qualitative research methods and Systematic reviews | Better implementation guidance for infection preventionists and healthcare epidemiologists during PHE. |
|  | What can we learn from organizations which did not experience negative trends in HAIs during the COVID-19 pandemic? | Infection preventionists must be an integral participant in disaster preparedness planning to better prepare facilities for future infectious disease pandemics. Although increasing beds during an emergency is part of disaster preparedness exercises, it isn't as common for the planning or exercise critiques to consider infection control issues. Since this may be a new role for IPs, determination of needed education, knowledge, and skills will assist in preparing IPs for this role. | Qualitive research methods and Systematic reviews | Inclusion of infection control concerns when planning for disasters. |
| 3. What is the impact of public health emergencies on the epidemiology of HAIs? | What can we learn from organizations which did not experience negative trends in HAIs during the recent covid pandemic? | Providing facilities with methods or practices that have worked can decrease patient mortality and staff burnout. | Qualitative research | Decrease time needed to adopt strategies that reduce morbidity and mortality |
| **5. How to Use Data to Prevent HAIs** | | | | |
| 1. Best study designs and statistical methods | What are the best study designs and statistical methods for data-driven hospital epidemiology? | Randomized trials are often prohibitively expensive and may not address all urgent questions | Increased focus on the use of new trial designs as well as observational techniques (e.g. causal inference methods) specific to the hospital epidemiology context | Increased body of appropriate methods for use in understanding infection transmission and evaluating interventions |
| 2. Risk assessment models for predicting harm | Which risk assessment models can be used to predict patient harm? | Risk assessment models need to be valid and robust, and applicable outside highly specialized academic medical settings | TRIPOD (transparent reporting of a multivariable prediction model for individual prognosis or diagnosis) reporting guidelines and an increased emphasis on validation and the use of generally available predictive variables | Generalizable, robust prediction models that are valid over a large number of clinical settings – including those not in the original model |
| 3. Data and analyses in providing information to clinicians | How can data and analyses consistently provide clinically significant and actionable information to clinicians? | Data analysis, no matter how sophisticated, that does not achieve clinical use does not improve patient outcomes | Validation and follow-up studies of risk prediction models, decision support tools, etc. that monitor clinical uptake.  The use of interactive notebooks, dashboards, etc. that make advanced analytical results accessible to a broader clinical audience | Increased acceptance and use of data-driven analysis and tools in clinical care |
| **6. Role of the Environment** | | | | |
| 1. Defining reservoirs in the healthcare environment | 1a. What known or novel reservoirs of epidemiologically important pathogens lead to healthcare facility outbreaks or ongoing risk of HAI? In particular, what risk is associated with the following: water sources, floors, surfaces, reusable shared medical equipment, shared spaces (e.g., gyms, waiting areas)? | Understanding where and how pathogens are harbored in the environment will assist in developing mitigation strategies. | Epidemiologic investigations of outbreaks with environmental cultures to demonstrate relatedness.  Environmental surveillance studies that assist in efficiently identifying sources of pathogens in the healthcare environment. | Information that leads to development of strategies to control pathogens in healthcare facilities. |
|  | 1b. What is the role of wastewater surveillance to detect outbreaks or risks to patients in healthcare facilities? | Wastewater sampling has been used to monitor viral disease epidemics and may be useful in identifying risks or mitigation strategies for other types of pathogens. | Measure association of wastewater pathogens with epidemiologic surveillance data in healthcare facilities. | Determine if wastewater surveillance provides timely or useful information to inform healthcare infection prevention strategies. |
| 2. Role of the healthcare environment in patient infection | 2a. To quantify the risk of environmental transmission, including risk of acquisition and infection | Understanding the relative contribution that environmental surfaces serve in healthcare associated infection will guide resource allocation | Prospective cohort studies to determine transfer of pathogens from environmental surfaces to patients. Randomized trials of cleaning interventions that include patient infection outcomes | Determine how often patients are infected by pathogens from the healthcare environment |
|  | 2b. What patient and patient-care risk factors predict acquisition of pathogens from the environment | Identifying risk factors for acquiring environmental pathogens may improve focused deployment of environmental cleaning resources | Prospective cohort studies to determine transfer of pathogens from environmental surfaces to patients. | Identify high-risk patients or care practices that may result in environmental transmission |
| 3. Strategies to reduce environmental contamination | 3a. What is the optimal implementation strategy to ensure adherence to thorough cleaning and disinfection of the healthcare environment? | Multiple studies have shown that thoroughness of cleaning is often inadequate and/or poorly sustained | Implementation studies using behavioral science techniques to optimize adherence to effective and thorough cleaning of the healthcare environment. | Recommended practices to improve and maintain the thoroughness of environmental cleaning and disinfection. |
|  | 3b. How to reduce contamination of environmental surfaces prior to terminal room cleaning? | Contaminated surfaces may play a role in transmission of pathogens through mobile equipment or HCP who interact with this environment prior to terminal room cleaning. | Cohort studies in order to identify patient and patient care features that result in contamination of surfaces. Interventional trials focused on reducing patient contamination of surfaces. Randomized trials of novel technologies such as continuous/prolonged surface disinfectants. | Practical solutions to reduce contamination of surfaces during patient occupancy. |
|  | 3c. What mitigation strategies are effective at controlling sources of opportunistic pathogens of premise plumbing or drug-resistant gram-negative pathogens in water sources (e.g., sinks, plumbing, ice machines), as compared with standard strategies? | Multiple healthcare outbreaks have been attributed to reservoirs of pathogens in water sources. We have an urgent need for strategies to interrupt transmission from water sources to patients, and/or control the pathogen burden harbored in healthcare plumbing. | Simulation studies of droplet transmission associated with standard vs. novel engineering solutions for sinks.  Studies demonstrating the efficacy of disinfection, mitigation, removal of biofilms, or reduced bioburden in healthcare facility sinks and plumbing. | Identification of mitigation strategies for water source HAIs. |

## Table 3: HCP wellness and burnout, device-associated infections, and the role of the community in the spread of infections in healthcare

| **Priority Area** | **Research Question** | **Clinical Rationale** | **Suggested Methodology/**  **Population** | **Intended Outcomes** |
| --- | --- | --- | --- | --- |
| **7. Healthcare Personnel Wellness and Burnout** | | | | |
| 1. Infection control healthcare workforce resiliency | 1a. How can flexibility be built into the infection control workload, and what is the best way to prioritize tasks? | The infection control workforce has been strained over the past several years with pandemic and non-pandemic related tasks. | Healthcare economics methods that assist with weighing the value of different interventions, such that decisions can be made about essential and non-essential tasks, are strongly encouraged. | Creation of a hierarchy of tasks, contingency plans, and back-up plans.  Identification of other HCP who can contribute during times when workload exceeds work force. |
|  | 1b. What are factors that support resiliency? | Challenges with credibility and politicized issues may have contributed to the perception that infection control efforts are not appreciated. | Qualitative investigations regarding perceived benefits versus the time required to complete different infection prevention tasks are also supported. |  |
|  | 1c. How do outside influences and feedback impact resiliency? | Identifying factors that contribute to job satisfaction and the perception that efforts are appreciated may help to promote long-term resiliency in the infection control work force. | Identifying factors that support resiliency, including career goals and satisfaction, using qualitative, quantitative, and mixed methods, and identifying strategies to leverage factors that encourage resiliency are encouraged. This may include interviews and surveys from infection control personnel and personnel working outside or collaboratively with infection control personnel. Implementation science frameworks may be useful guides for identifying contextual factors that should be considered when evaluating resiliency. |  |
| 2. Sustainability of the IC healthcare workforce | 2a. How can we create a sustainable workforce that can adapt to changing workloads and circumstances? | The nature of infection control practice is that many infection control programs have FTE to support day-to-day requirements.  There is limited capacity to take on new projects when emergencies occur.  Evidence from other fields, such as Veterinary medicine, suggests that the provision of futile care and administrative tasks contribute substantially to burnout.  The electronic health record is often cited as a driver of burnout. Do these same factors contribute to infection control? How can these factors be mitigated? | Identification of effective mentoring, collaboration, and outreach strategies to recruit into the field are encouraged.  Questions of sustainability can be addressed through various mechanisms, including healthcare economics and implementation science methods.  Regulations and policy changes necessitating a high administrative burden may be factors that contribute to burnout. Identification of technological strategies that can limit administrative burden, such as fully electronic surveillance, may effectively limit burnout.  Identifying specific technological strategies coupled with assessments of how they impact workflow, load, and perception of workload are critical for identifying how interactions with the electronic health record can be optimized.  Identifying strategies that professional organizations can leverage to recruit to the specialty. | Identifying strategies to build “slack” into the infection control workforce and describe and identify sustainable funding and FTE models to better allow for emergency responses.  Identify factors (e.g., specific tasks) in infection control that drive burnout and develop an evidence base to address them (e.g., identify effective strategies for reducing the impact of factors that drive burnout, such as automation of surveillance activities).    Collaboration with other organizations, such as APIC, is recommended. |
|  | 2b. How can we recruit into the specialty? |  |  |  |
|  | 2c. What aspects of the infection control workflow contribute the most to burnout, and what factors mitigate burnout? |  |  |  |
|  | 2d. What factors lead individuals to leave the specialty? |  |  |  |
|  | 2e. How can these factors be addressed in an evidence-based way to support the ongoing health and well-being of the healthcare workforce? |  |  |  |
| 3. Identification of novel communications strategies | 3a. How can we improve relationships with stakeholders during emergency and non-emergency situations? | Ongoing communication with stakeholders to promote good infection prevention practices is critically important for sustaining quality | Multi-disciplinary investigations with experts in different fields, including implementation science, health communications specialists, psychologists, behavioral scientists, and anthropologists are encouraged. | Creation of new models of evidence-based communications strategies that can be used across various infection prevention interventions |
|  | 3b. What are the most effective health communications strategies to improve the adoption of infection control best practices? | Developing relationships outside of emergency situations fosters positive interactions when stresses on the system occur | New evidence-based models of health communications and behaviors are needed that can be used as a guide to creating communications materials. | Identification of effective stakeholder engagement and communications strategies |
|  | 3c. What are the best health communications strategies for interacting with hospital stakeholders, peer organizations, policymakers, and the public? | Little is known about the best practices for promoting the adoption of evidence-based infection prevention strategies | Mixed methods studies that include quantitative and qualitative elements to identify factors that drive dissemination and diffusion of evidence-based and non-evidenced based information are strongly encouraged to enhance our understanding of how information and mis and disinformation spreads. | Identifying similarities and differences in effective communication for different audiences (e.g., peer organizations, hospital leadership, HCP, and the public) |
|  | 3d. How should messaging vary depending upon the intended audience? | Health communications literature suggests that knowledge and education are necessary but not sufficient. | Identify strategies to improve the speed and scope of uptake evidence-based interventions and to de-implement ineffective interventions. The randomized controlled trial comparing different vaccine educational strategies and intention to vaccinate children is a model that could be followed to test effective communications interventions empirically. | Identify implementation and communication strategies that are effective for sharing and disseminating information.  Different contexts may require different messages and communications strategies may need to be tailored and individualized. Strategies to identify best practices based on target audience and context are encouraged. |
|  | 3e. How is information shared and circulated? | Best communications strategies may vary depending upon the intended audience |  |  |
| **8. Device-Associated Infections** | | | | |
| 1. Surveillance targets and definitions | 1a. What is the lowest achievable frequency of a given device associated infection? | In practice not all infections are necessarily preventable. Thus, the goal of “0” HAI may be an unachievable ideal that encourages inefficient resource allocation. | Systematic literature reviews of observational intervention studies have been used to estimate the proportion of potentially preventable infections. Prospective studies in well controlled settings with a high level of adherence to best practices could estimate the lowest achievable infection rates. | A practically achievable low-end target for preventable infections can help infection prevention programs target infection reduction efforts where benefit is greatest. |
|  | 1b. What patient outcomes are associated with different categories of HAI events? | Estimates for the morbidity and mortality associated with HAI events need to be updated when surveillance definitions change. | Retrospective analysis of patient outcomes such as mortality, length of stay, ICU length of stay, ventilator dependence, that are associated with HAI events (e.g., outcomes associated with each category of ventilator associated event) | Estimates of morbidity and mortality associated with HAI events help to prioritize infection prevention activities and support the need for resource allocation for such activities. |
|  | 1c. Which HAI reduction interventions achieve the greatest improvement in patient care and outcomes? | The field of infection prevention should prioritize interventions that improve patient clinical outcomes. | Prospective and randomized trials of infection prevention interventions that report patient care process measures and clinical outcomes (ex, mortality, functional impairment, length of stay) | Identify patient-centered infection prevention interventions that improve patient care and clinical outcomes and estimate the amount of improvement. |
| 2. Novel device technologies and maintenance strategies | 2a. Do non-invasive devices (or management strategies) adequately support patient care and reduce HAI risk? | Avoidance of invasive devices reduces the risk of device-related infection. | Comparative, randomized trials of non-invasive technologies (or management strategies) versus standard invasive devices in care of complex patient populations. | Provide evidence that non-invasive technologies (or management strategies) are 1) effective in supporting patient care and 2) safer than invasive devices. |
|  | 2b. Do novel device technologies (or maintenance strategies) reduce HAI risk as compared with standard devices? | Innovative designs for invasive devices (or maintenance strategies) aim to reduce the risk of infection. Examples may include colonization-resistant catheters, biofilm-resistant substrates, or devices that provide mechanical or biological biofilm disruption. | Comparative, randomized trials of novel invasive device designs (or maintenance strategies) versus standard of care among patients who require invasive device use. Outcomes should include patient-focused outcomes (e.g. HAI), unintended consequences (e.g. acquired drug-resistance, device malfunction), and costs. | Provide evidence that novel technologies are 1) effective in supporting patient care, 2) safer, 3) worth resource investments as compared with standard of care. |
| 3. High-risk populations | 3a. Define the incidence of device-associated infections in outpatient settings | Establishing the epidemiology of infections outside of acute care will aid in development of prevention strategies | Cohort studies of patients with invasive devices outside of the acute care setting | Provide the incidence of device-associated infection in these populations, potential risk factors, and clinical outcomes |
|  | 3b. Identify strategies to reduce central line-associated infection in dialysis patients | Unique strategies are needed in patients with prolonged venous access catheter use and frequent healthcare exposure | Randomized trials of both novel devices and non-invasive strategies to reduce MDRO colonization and central line infection | Develop practical strategies to reduce device-associated infection that may be applied in both inpatient and outpatient dialysis settings |
|  | 3c. Identify strategies to reduce central line-associated infection in oncology patients | Immunosuppression, mucosal barrier injury, and skin diseases result in high rates of bloodstream infections, including CLABSI | Cohort studies focused on identifying risk factors for mucosal barrier injury laboratory confirmed bloodstream infection (MBI-LCBI) and CLABSI. Interventional trials focused on reducing CLABSI in patients with prolonged catheter use, including after stem cell transplantation | Provide specific guidance to reduce MBI-LCBI and CLABSI in oncology patients. |
| **9. The Role of the Community in the Spread of Infections in Healthcare** | | | | |
| 1. Epidemiology of HAIs in the community | 1a. Which community settings encounter or contribute to HAIs? | Very little is known about the burden of HAIs in the community. Free-standing ambulatory settings not affiliated with a hospital or health system may not have access to trained HE/IPC staff. For-profit clinics may not see infection prevention as a priority, particularly when there are no reporting requirements. Lack of adherence to aseptic technique and instrument reprocessing guidance may result in outbreaks. | Population cohort or point-prevalence studies in various community healthcare settings.  Community healthcare setting surveys across care settings including long term care, allied health clinics (e.g., dental), medical/surgical clinics, infusion centers, procedural clinics, home health and patients at home, | HAI prevalence (CLABSI, CAUTI, SSI, CDI, MDRO), setting-specific  Infection prevention practices across care settings |
|  | 1b. How should one determine that an infection is healthcare-associated? Which types of HAIs are associated with which settings? | Because of limitations in post-discharge surveillance, most HAIs are currently identified through office visits or readmissions, which often misses less severe cases. However, for a few settings and infection types, the CDC NHSN has surveillance criteria for determining if the infection was healthcare-associated. | Conduct literature review to identify existing guidance and gaps. Convene an expert panel to develop consensus-based guidance on determining if an infection is healthcare-associated. | Surveillance systems with standardized definitions  Accurate and reliable algorithms for calculating numerators and denominators |
|  | 1c. What is the extent of antimicrobial resistance among HAIs in the community? | Without routine laboratory surveillance mechanisms, it is difficult to determine the extent of AR among HAIs in the community. Similarly, it is challenging to distinguish between community-associated infections and HAIs (ref Alsaedi 2022, CDC communities). | Engage with CDC’s Antibiotic Resistance Laboratory Network (AR Lab Network), established in 2016, provides nationwide lab capacity to rapidly detect antibiotic resistance and inform local responses to prevent spread and protect people ([CDC AR Lab Network](https://www.cdc.gov/drugresistance/laboratories.html))  Point-prevalence survey of HAI/MDRO prevalence in community settings using private insurer or Medicare/Medicaid claims-based data | MDRO, *C. difficile* prevalence  CLABSI, CAUTI, SSI, MDRO, *C. difficile* prevalence |
| 2. Surveillance in community healthcare settings other than hospitals | 2a. What are effective strategies for performing HAI surveillance in non-hospital settings? To what extent can syndromic surveillance of voluntarily shared EMR data be used to reliably identify HAIs for outpatient care (ED, urgent care, office-based surgery)? | Most non-acute care hospital settings (e.g. psychiatric and long-term care hospitals), nursing homes and home care organizations do not have standardized HAI surveillance and reporting systems. The exception to this is Medicare-certified dialysis centers and certain types of ambulatory surgery centers where reporting to the CDC NHSN is required ([CDC NHSN Dialysis](https://www.cdc.gov/nhsn/dialysis/index.html)). | Surveillance of community-based labs in partnership with public health (e.g., mandated lab-based reporting).  Point-prevalence survey of HAI/MDRO prevalence in community using private insurer or Medicare/Medicaid claims-based data. Qualitative research with business leaders and frontline staff across settings and companies. Formal improvement collaboratives are effective for increasing use of existing tools and resources. | MDRO, *C. difficile* prevalence  CLABSI, CAUTI, SSI, MDRO, *C. difficile* prevalence  Enhanced surveillance of community settings.  Less transmission of infections. |
| 3. Barriers to infection prevention in community settings | 3a. How can HE/IPC staff assist in training staff in non-hospital settings? What training is needed? What are effective strategies for conducting the training? Which staff in which settings should be trained? How often is training needed, considering the rate of staff turnover? | Some community healthcare settings have no staff with HE/IPC expertise. IPC responsibilities are often delegated to staff who lack IPC training and support. | Qualitative needs assessment survey to develop or adapt existing training strategies and modalities to meet the needs of frontline staff. Consider cultural and language needs. | Easily accessible and appropriate training modalities. |
|  | 3b. How can HE/IPC staff assist non-hospital settings overcome equipment, supply, and physical resource constraints in non-hospital settings? | Many outpatient healthcare settings lack room for separating clean and dirty areas. Similarly, they often struggle to obtain PPE for staff and lack cleaning supplies and staff to adhere to guidance. Some settings that need to perform HLD and sterilization of medical instruments (such as dental, plastic surgery, and other ambulatory surgical settings), struggle with adequate space, preventative maintenance, and training on HLD/sterilization processes. | Survey of ambulatory care setting resources |  |
|  | 3c. For community congregate living settings that are not licensed HCP (e.g., jails, senior housing, etc.), what are their expectations for infection prevention? | There exists very little IPC guidance for staff in community settings that are not primarily healthcare but have increased risk for outbreaks due to congregate living arrangements | Convene meetings of HE/IPC experts together with facility staff to identify needs and practical solutions relevant to the specific settings. | Established monitoring systems  Less transmission of infections. |
| 4. Models for Effective Infection Prevention in Community Settings | 4a. What are effective strategies for HCP-based infection prevention in various community healthcare settings? | Infection prevention expertise and resources are severely limited in community healthcare settings. Little is known about effective and practical strategies that can be implemented to prevent HAIs in these settings. | Observational prospective pre/post cohort studies or cluster-randomized trials evaluating the impact of IP strategies specific to different community healthcare settings | Reductions in HAIs/MDROs.  Compliance with infection prevention practices – HH in clinics, CHG bathing (e.g., for surgical patients) |
|  | 4b. What are effective strategies for patient-based infection prevention in various community healthcare settings? | Patient engagement in infection prevention at home or within healthcare settings is understudied. | Patient-based survey  Observational prospective pre/post cohort studies or cluster-randomized trials assessing the impact of patient-based education, telemedicine, CHG-bathing, or other self-administered IP activity. | Knowledge, attitudes, barriers to effective infection prevention in the home.  Decrease in HAIs/MDROs |

## Table 4: The Role of Social Interaction and Engagement for the Public, Patients, and Visitors, Occupational Safety

| **Priority Area** | **Research Question** | **Clinical Rationale** | **Suggested Methodology/**  **Population** | **Intended Outcomes** |
| --- | --- | --- | --- | --- |
| **10. The Role of Social Interaction and Engagement for the Public, Patients, and Visitors** | | | | |
| 1. Behavior change strategies | 1a. What are the emotional, physical and safety burdens to the patient? | Since the burden of isolation is largely placed on the patient, we should know if there is measurable harm to the patient when placed on TBP. | Surveys of satisfaction, emotional state and measures of harm comparing matched patients not on isolation.  Special population: Long Term Care | Measured relative safety and emotional consequence of isolation |
|  | 1b. How effective are the isolation practices in reducing cross-infection? | The value of isolation changes with levels of transmission outside healthcare. Studies that rely on before and after rates may mistake temporal trends for efficacy. | Interrupted time series and step-wedge comparisons should be carried out and repeated. Establishing compliance with isolation can help validate efficacy or lack thereof. | Relative risk reduction in cross-infection using isolation. |
| 2. Visitors | 2a. Do visitation restrictions change the incidence of healthcare associated infections? | Though pathogens sometimes enter healthcare through visitors, the contribution of visitor restriction to HAI reduction is unknown. | Interrupted time series and step-wedge comparisons of policy changes and HAI rates. | Identification of what restrictions provide quantifiable benefit |
|  | 2b. What are the emotional, social, and physical costs to the patient from visitor restriction? | Patients’ satisfaction, emotional well-being and care are enhanced through their family and friends. The burden on patients and visitors is not well delineated. | Evaluation of patient outcomes and well-being as a function of visitation restrictions. The quantity of visitors should be measured since some patients receive more visitors than others. | Establish metrics to guard against patient harm during visitor restrictions. |
|  | 2c. Does limiting access to visitors meaningfully prevent the transmission of pathogens? How can visitors be best protected from pathogens when they visit? | Hospitals provide safety measures for HCW providing care. These safety measures can be used by visitors. | Clarify the epidemiology of pathogen movement into and out of the healthcare environment, supported by molecular methods. Determine the extent of community prevalence of pathogens to inform visitor restrictions. | Understand the movement of pathogens between the public and healthcare. Balance the least costly burden to patients and families with public good with |
| 3. Role of Patients in the Prevention of Infection | 3a. What role do patients play in infection prevention? | Prevention of HAIs is largely the responsibility of healthcare. However, patient factors may also influence the outcome. | Controlled trials of patient education and prevention efforts with HAI as outcome measures.  Developing measures of compliance with education to confirm correlation with effectiveness. | A set of established best practices for patients. |
|  | 3b. Do patient reported outcomes reliably reflect safety. | Patient satisfaction is often used as a measure of quality. Though the patient’s experience of care is at the critical point of contact, the tools used can be influenced by many factors. | Reviews of PSOs across a broad population compared to more objective quality metrics. | PSOs that reflect desirable outcomes that supplement HAI harm measures. |
| 4. Communication with the Public and Patients | 4a. How do we know our message is having the desired effect? | There are many ways that the message can be shaped. Which method of messaging these methods is best able to result in changes in knowledge or HAI outcomes? | Separate communication methods from other interventions to determine changes related to the communication (e.g. cluster-randomized trials). | Linking communication to effect. |
|  | 4b. Do publicly reported metrics communicate a useful message to the public? | In theory, quality and safety metrics should drive consumers' choices. The evidence that public comparison sites influence behavior is unclear. Many metrics lag current performance by several years. | Survey the public on understanding of different methods of presentation.  Establish which metrics reliably correlate with future performance. | Clear metrics that are understood by the public that reliably reflect current quality of outcomes. |
| **11. Occupational Safety** | | | | |
| 1. What strategies are needed to optimize occupational health protections, including during public health crises and incident management | 1a. What are best practices for response and how do they vary by type of disaster? How should they be identified / discovered, evaluated according to criteria, and disseminated? | A better understanding of preferred practices is needed to improve the effectiveness of HCP’s response and minimize negative outcomes for patients, staff and community members. | Qualitative research, case studies across varied locations and events, evaluated by experts and widely disseminated. | Improved response to emergencies with a standardized measure to track specific components of the response practices |
|  | 1b. To what extent are occupational health leaders engaged in emergency preparedness planning and drills etc. at the organization or system level? How do IPC/HE (infection prevention and control/healthcare epidemiology) and Occupational Health engage in collaborative planning? | Occupational health leaders should be engaged in planning and scene response to employ effective strategies for emergencies. IPC/HE staff should be confident that Occ Health can support common needs/goals (AAOHN 2013). As described by Rogers 2007, occupational health nurses and occupational and environmental medicine physicians require not only clinical skills related to illness and injury but also skills in surveillance, management, community coordination, risk management and communication. These skills often overlap with HE/IPC. | Survey members of relevant professional associations; qualitative interviews. Because of the nature of these activities, it is unrealistic and impractical to do clinical trials. | Expectations for Occupational Health’s role in preparedness  Increased awareness of the complementary skills between Occupational Health and IPC/HE to enhance their roles in preparedness  Better prepared staff to carry out needed responses during events |
|  | 1c. What are the minimum expectations for PPE stockpiles within organizations? Which types of items and how many of each should healthcare organizations have on hand before emergencies hit? Can PPE be used safely for some time after their expiration date in times of emergency? What storage requirements are needed? Can we establish a national (? Regional?) database of PPE stockpiles in organizations so that we can share resources effectively during crises? | Immediate access to PPE is necessary in emergencies. However, there are no national or international standards and little guidance on PPE stockpiling within healthcare organizations (Greenwald 2021, Wizner 2016) Some facilities rely on tight supply chains for just-in-time procurement while others maintain supplies that may expire or become obsolete. A transparent system for sharing information on stockpiles is needed. | Convene government and academic experts to establish local and national recommendations.  Assess adherence to recommendations using on-site visits or via a questionnaire. | Greater local and national confidence in preparedness with easily accessible, trackable stockpiles to better support staff and patient safety in an equitable way against infectious pathogens. |
|  | 1d. When settings lack occupational health physicians or staff, how should IPC/HE staff best provide just-in-time (JIT) training on needed competencies? Can we leverage virtual platforms for such training and consultation when needed? Can simulation training be effectively used? | Examples of settings often lacking staff trained in occupational health include nursing homes, assisted living centers, behavioral health centers, free-standing dialysis centers, surgical centers, urgent care, office-based settings and home health care | Assess adherence to IPC/HE practices in these settings.  Conduct qualitative studies to understand current knowledge and perceived barriers to target future interventions for JIT training.  Evaluate the effectiveness of simulation training for emergency preparedness | JIT training modules that are easily accessible to staff in non-traditional medical settings. |
|  | 1e. Given the societal trend toward pushing back against mandatory vaccination (including influenza), how should facilities balance vaccine requirements without jeopardizing staffing levels? | With growing misinformation and disinformation that is increasingly mainstream, IPC/HE leaders need up-to-date strategies to optimize safety against vaccine preventable infections while recognizing staffing constraints. | Conduct qualitative studies to assess HCP attitudes and beliefs towards vaccines | Improved HCP education and adherence to national guidance surrounding vaccinations and thereby improved employee safety |
| 2. Role of the HCP in transmission | 2a. How should hospitals measure or conduct surveillance to evaluate the extent of presenteeism? | Presenteeism is associated with several adverse outcomes for patients, HCP, and the organization. Examples including HAI outbreaks, reduced productivity, and burnout in staff. As described by Albrich 2008, HCP can be the source, vector, and/or victim of infectious diseases. | Compare accuracy of two or more measurement approaches, ideally standardized across multiple sites | Understanding the prevalence of presenteeism may help researchers better identify incentives and approaches to decrease this ongoing practice, hence improving patient and staff wellbeing. |
|  | 2b. How effective are local or national policies requiring daily screening or testing of healthcare staff? What are the pros and cons of passive daily self-screening with or without reminders versus active testing during outbreaks? How does the surveillance approach affect staffing levels? | Countries that adopt strict work restriction policies may experience staffing shortages and reductions in services. Testing practices, when tied to meager sick leave policies, can deplete staff’s paid time off. In the US, surveillance strategies vary across sites but there is little information on most effective practices. | Examine associations between various symptom screening and pathogen testing approaches and staff shortages, staff burnout, HAI outbreaks of tracked pathogens, and patient access to care | Evidence-based guidance on local and national practices for optimal sick symptom/testing practices for HCP as well as standardized tracking processes. |
|  | 2c. What is the role of Occupational Health and IPC/HE in establishing and implementing organization-level policies for managing sick leave to promote patient/staff safety and decrease presenteeism? | There is great variability across hospitals in both the content of the policies and how they are implemented that may have significant impact on staff presenteeism. | Survey occupational health and human resources professionals to understand policies.  Survey IPC/HE staff to understand role and perceptions of influence and effectiveness in policy development and implementation | Understanding current practices for sick leave provides occupational health professionals the opportunity to advocate for best practice in HCP. |
|  | 2d. What data collection systems are needed to be research ready for emergencies across systems, states, national databases. What are effective ways to plan for surges that would accommodate staff becoming ill? | Currently there is no national source of staffing information in hospitals that could support sharing across locales etc. Understanding staffing availability would support preparedness planning and help decrease presenteeism. | Establish a national, standardized mandatory reporting system for staffing information in acute care (as existed during COVID in nursing home care). Integrate into existing reporting systems when possible | Easy access to local sources for staff when needed in emergencies |
|  | 2e. What is the role of organizational culture, leadership and programs to support wellness? How can they be used to reduce presenteeism (and encourage appropriate absenteeism)? What are examples of best practices? | Studies have shown that hospitals with leadership and organizational cultures that support staff wellness have greater staff engagement, less turnover, less distress and burnout among employees when under emergency situations (Melnyk 2022, Brand 2017) | Determine how much performance on widely-used safety culture measures (e.g., AHRQ Hospital Survey of Patient Safety) correlates with staffing levels and/or presenteeism and staff perceptions of stress. | Improved staff resilience and well-being while decreasing presenteeism, burnout, and turnover. |
|  | 2f. How should we address the widespread belief among staff that it is better to work when sick than to call in? | HCP often have strong sense of duty to both patients and colleagues; they feel guilty because calling-in sick increases burden on other staff, nor do HCW want to appear weak (sidenote – same issue with taking breaks and vacations) (Szymczak 2015). As described by Tartari 2020, the decision to work through illness is a complex phenomenon shaped not only by personal factors or lack of knowledge regarding the importance of the risk of transmission but, more importantly, by the organizational culture and policies with financial penalties, which may place possible constraints on absenteeism, thus resulting in presenteeism. | Identify, examine and share examples of effective practices that change beliefs.  Conduct studies that compare outcomes for patients, HCP, and organizations with high versus low presenteeism | Understanding of the mental model behind presenteeism in order to decrease its practice in the healthcare setting. |

## Table 5: Post-Acute Care and Nursing Homes, Pediatrics

| **Priority Area** | **Research Question** | | **Clinical Rationale** | **Suggested Methodology/**  **Population** | | **Intended Outcomes** | |
| --- | --- | --- | --- | --- | --- | --- | --- |
| **Post Acute Care and Nursing Homes** | | | | | | | |
| 1. Interventions to reduce/prevent healthcare associated infections (HAIs) and transmission of multi-drug resistant organisms (MDROs) | How can we develop, implement and sustain practical interventions to reduce common HAIs and transmission of MDROs in post-acute and nursing home settings using available or limited resources? | | High quality evidence is necessary to design and implement interventions to reduce HAIs, inappropriate antibiotic use and transmission of MDROs in nursing homes. | Patient-level randomized controlled trials, facility-level cluster-randomized and stepped-wedge studies | | Strong evidence that is practical and deployable in resource limited settings where older adults with multiple risk factors and comorbidities are cared for | |
| 2. Evaluate the role of environmental contamination in the transmission dynamic of pathogens | What role does environment play in transmission of viral and bacterial pathogens? | | It is critical to understand the role of environmental contamination in the chain of transmission of pathogens, especially MDROs | Prospective, longitudinal studies that integrate pathogen-, patient-, nursing home-level information and utilizing innovative and newer molecular and genomic techniques | | In depth studies defining epidemiology of environmental contamination with various viruses and bacteria, their duration of contamination, correlation with patient strains and frequency of transmission to HCP and patients | |
| 3. Collaborative models between nursing homes and local hospitals and other state health authorities | What models of collaboration between nursing homes and other healthcare entities help to promote the open exchange of information, evidence and strategies to reduce infections and make transfers as well as care delivery safer while keeping patients and families engaged? | | It is important to support infection prevention training and practices in post-acute and nursing home settings to promote rapid responses to infectious outbreaks and pandemics. | Implementation science methods, mixed methods, quasi-experimental and stepped wedge-designs | | Develop simple, systematic models of collaboration between nursing homes and other regional healthcare entities, support nursing home staff with resources and access to experts. | |
| **Pediatrics** | | | | | | | |
| 1. What Are Effective and Sustainable Practices to Decrease Pediatric HAIs? |  |  | | |  | |  |
| 2. What is the impact of visiting policies and related infection control measures on in-hospital transmission of viral illnesses in the pediatric/neonatal settings? | 2a. How common is transmission of respiratory and/or gastrointestinal viral infections from visitors to patients in acute, post-acute, and long-term pediatric care settings? | There is tension between the need for caregiver presence at bedside for hospitalized children and the risk of exposing patients to viral illnesses. It is therefore critical that high-quality research informs best practices surrounding visitation policies to balance these priorities. | | | High-quality studies including those utilized quasi-experimental or randomized designs are well-suited to evaluating the impact of various approaches. | | Identify the role of visitors in transmission of healthcare-acquired viral infection. |
|  | 2b. What is the more effective approach to visitor restriction policies: year- round or seasonal policies? |  |  |  | Measurement of multiple outcomes should be prioritized, including implementation (e.g., acceptability, adherence), patient and family-centered (e.g., caregiver presence for rounds), effectiveness (e.g., rates of healthcare-acquired viral infections), and harms/downsides of the approach. | | Compare optimal timing of visitor restriction/screening policies and weigh benefits and potential downsides of the strategy. |
|  | 2c. What are the harms of restricted visitation strategies? |  |  |  |  |  | Identify contexts in which the strategy is appropriate or inappropriate. |
|  | 2d. Are there settings in which visitor restrictions should not be applied? |  |  |  |  |  | Evaluate best practices to reduce the spread of viruses in healthcare settings, including visitor hand hygiene measures and mask use. |
|  | 2e. What are the most effective strategies to minimize transmission from visitors to patients (e.g., hand hygiene, masking)? |  |  |  | Studies should be conducted across the spectrum of pediatric inpatient settings, including acute care hospitals, as well as post-acute care facilities. | |  |
| 3. What is the prevalence of non-device associated HAIs in children (including respiratory viral infections)? | 3a. How common is transmission of respiratory viral infections in healthcare settings? | Respiratory viruses are common in children, and many respiratory viral infections in pediatric populations may be asymptomatic. | | | Genomics studies that definitively link epidemiologically associated cases are critical for advancing knowledge about frequency and nature of spread. | | Identify the frequency at which respiratory viral infections are transmitted in pediatric healthcare settings, and quantify risk of transmission based on setting, pathogen, and host. |
|  | 3b. How do rates differ according to patient, pathogen, and clinical setting (e.g., inpatient versus outpatient)? | Little is known about the nature of respiratory viral transmission in inpatient and outpatient pediatric healthcare settings. | | | To expand our understanding about prevalence and natural history, investigations should include control groups and careful denominator assessment. | | Improve understanding of the real-world clinical and economic effectiveness of different infection prevention interventions designed to reduce transmission. |
|  | 3c. How common is asymptomatic transmission? |  |  |  | Case reports and singular outbreak investigations do not provide an accurate view of prevalence and are specifically discouraged. Extrapolation of data from one respiratory virus and applied to other respiratory viruses are also specifically discouraged. | | Identify interventions that are sustainable in the long-term to inform policy about how, when, and for how long different interventions are useful. |
|  | 3d. What interventions are effective for preventing the transmission of respiratory viruses in healthcare settings? | Data are needed to inform effective infection prevention policy to prevent the spread of respiratory viral infections in healthcare settings. | | | Experimental and quasi-experimental designs are encouraged to evaluate the effectiveness of different interventions for controlling spread. | |  |
|  | 3e. How does context (patient, pathogen, clinical setting) impact risk? |  |  |  | Implementation considerations, such as context, should also be considered in any evaluation and effectiveness and relative effectiveness in different settings measured. | |  |
|  | 3f. How well does evidence generated in laboratory and experimental settings translate to real-world infection prevention? |  |  |  | Data about inpatient and outpatient settings of care are important for informing infection prevention practice. | |  |
|  |  |  |  |  | Economics evaluations that consider return on investment and risks and benefits of different prevention strategies are also encouraged. | |  |
| 4. How to improve the definitions of HAIs for neonatal populations? |  |  |  |  | Implementation investigations that measure and inform the long-term sustainability of interventions are also needed. | |  |
